# Supplementary material for: Integrating hydrogen utilization in CO2 electrolysis with reduced energy loss
Source: Nat Commun. 2024 Feb 16;15:1427. doi: 10.1038/s41467-024-45787-x (PMC10873292; doi:10.1038/s41467-024-45787-x)
Supplement: Supplementary file 1 — Supplementary Information [file 41467_2024_45787_MOESM1_ESM.pdf]

Supplementary Information

Integrating hydrogen utilization in CO_2 electrolysis with reduced energy loss

Xiaoyi Jiang[1,2], Le Ke[1,2], Kai Zhao[1,2], Xiaoyu Yan[1], Hongbo Wang[1], Xiaojuan Cao[1], Yuchen Liu[1,2], Lingjiao Li[1,2], Yifei Sun[3], Zhiping Wang[1], Dai Dang[4], Ning Yan[*1,2]

[1]School of Physics and Technology, Wuhan University, Wuhan, 430072, China

[2]Shenzhen Research Institute of Wuhan University, Shenzhen, 518057, China

[3]Shenzhen Research Institute of Xiamen University, Shenzhen 518057, China

[4] School of Chemical Engineering and Light Industry, Guangdong University of Technology, Guangzhou, 510006, China

*To whom the correspondence should be addressed: ning.yan@whu.edu.cn

This Supplementary Information includes:

1. Supplementary Tables……………………………………………………………..3

2. Supplementary Figures…………………………………………………………...24

3. Supplementary Notes………………………………………………………… 65

4. Supplementary References…………………………………………………….....68

1. Supplementary Tables

Supplementary Table 1. The comparation of thermodynamic data between conventional and H_2-integrated CO_2RR at standard conditions.

reaction of conventional CO_2RR	Δ_rG (kJ mol^{-1})	E_0 (V)	reaction of H_2-integrated CO_2RR	Δ_rG (kJ mol^{-1})	E_0 (V)
$CO_2 + H_2O \rightarrow HCOOH + 0.5O_2$	257.18	1.41	$CO_2 + H_2 \rightarrow HCOOH$	34.95	0.18
$CO_2 \rightarrow CO + 0.5O_2$	272.09	1.33	$CO_2 + H_2 \rightarrow CO + H_2O$	20.04	0.10
$CO_2 + 2H_2O \rightarrow CH_4 + 2O_2$	818.12	1.06	$CO_2 + 4H_2 \rightarrow CH_4 + 2H_2O$	-130.45	-0.17
$2CO_2 + 2H_2O \rightarrow C_2H_4 + 3O_2$	1331.37	1.15	$2CO_2 + 6H_2 \rightarrow C_2H_4 + 4H_2O$	-91.48	-0.08

E_0 is the Nernst potential at standardsas conditions.

Supplementary Table 2. Mass loadings of Zn nanosheets on a 1×2 cm^2 Cu foam.

	$M_{Cu\ foam}$ (mg)	$M_{Cu\ foam\ +\ Zn}$ (mg)	M_{Zn} (mg)	Zn loading (mg cm^{-2})
Zn-Cu-100	129.54	139.31	9.77	4.89
Zn-Cu-500	116.56	134.02	17.46	8.73
Zn-Cu-1000	122.68	149.39	26.71	13.36

Supplementary Table 3. Fitting results of the EIS plot in Figure 2h.

Potential (V vs. RHE)	$R_{ct}(\Omega)$	$R_w(\Omega)$	C(F)
1.40	0.24	2.90	0.81
1.50	0.21	0.90	0.75
1.65	0.15	0.50	0.75
1.80	0.24	-	0.70

Supplementary Table 4. Fitting results of the EIS plot in Figure 2i.

DoC (%)	$R_{ct}(\Omega)$	$R_w(\Omega)$	C(F)
0	0.20	0.70	0.75
30	0.22	1.20	0.77
60	0.19	1.40	0.77
100	4.19	-	0.70

Supplementary Table 5. Fitting results of the EIS plot in Figure 2j.

Reaction	$R_{ct}(\Omega)$	$R_w(\Omega)$	C(F)
NiOR	0.20	0.70	0.75
OER-NiOOH	4.19	-	0.70
OER-Co_3O_4	2.45	-	0.84

Supplementary Table 6. Fitting results of the EIS plot in Supplementary Fig.15.

Potential (V vs. RHE)	$R_{ct}(\Omega)$	$R_w(\Omega)$	C(F)
1.25	0.20	1.18	0.68
1.15	0.23	1.90	0.87
1.05	0.25	4.30	0.90

Supplementary Table 7. The product FE, cell voltage, electrode potentials, and overpotentials (η) in combined CO_2RR+NiOR for CO production.

Current density (mA cm^{-2})	H_2 FE (%)	CO FE (%)	Cell voltage (V)	$Potential_{cathode}$ (V vs. RHE)	$\eta_{cathode}$ (mV)	$Potential_{anode}$ (V vs. RHE)	η_{anode} (mV)
20	24.4	70.6	1.82	-0.40	300	1.39	40
50	22.7	71.3	2.15	-0.52	420	1.53	180
70	21.9	73.1	2.30	-0.59	490	1.58	230
100	19.9	75.6	2.47	-0.66	560	1.62	270
120	19.6	76.4	2.59	-0.70	600	1.66	310
150	17.7	81.9	2.72	-0.74	640	1.70	350
200	18.1	78.5	2.91	-0.79	690	1.74	390
250	20.2	76.5	3.10	-0.85	750	1.78	430

Supplementary Table 8. The product FE, cell voltage, electrode potentials, and overpotentials (η) in combined CO_2RR+OER for CO production.

Current density (mA cm^{-2})	H_2 FE (%)	CO FE (%)	Cell voltage (V)	$Potential_{cathode}$ (V vs. RHE)	$\eta_{cathode}$ (mV)	$Potential_{anode}$ (V vs. RHE)	η_{anode} (mV)
20	28.6	65.5	2.01	-0.42	320	1.56	330
50	27.8	71.3	2.32	-0.52	420	1.70	470
70	27.2	72.5	2.50	-0.60	500	1.77	540
100	23.4	74.6	2.67	-0.66	560	1.82	590
120	22.7	76.4	2.76	-0.69	590	1.84	610
150	22.1	77.4	2.89	-0.73	630	1.87	640
200	19.3	76.4	3.06	-0.78	680	1.90	670
250	20.4	72.6	3.25	-0.84	740	1.94	710

Supplementary Table 9. The product FE, cell voltage, electrode potentials, and overpotentials (η) in combined CO_2RR+NiOR for formate production.

Current density (mA cm^{-2})	H_2 FE (%)	formate FE (%)	Cell voltage (V)	Potential$_{cathode}$ (V vs. RHE)	$\eta_{cathode}$ (mV)	Potential$_{anode}$ (V vs. RHE)	η_{anode} (mV)
20	10.2	87.8	1.90	-0.46	280	1.39	40
50	5.1	93.8	2.17	-0.54	360	1.53	180
70	5.2	94.8	2.30	-0.60	420	1.58	230
100	5.6	94.3	2.47	-0.70	520	1.64	290
120	4.0	95.1	2.53	-0.73	550	1.67	320
150	4.2	95.3	2.61	-0.75	570	1.70	350
200	4.3	95.0	2.81	-0.77	590	1.74	390
250	12.0	84.2	3.01	-0.79	610	1.78	430

Supplementary Table 10. The product FE, cell voltage, electrode potentials, and overpotentials (η) in combined CO_2RR+OER for formate production.

Current density (mA cm^{-2})	H_2 FE (%)	formate FE (%)	Cell voltage (V)	$Potential_{cathode}$ (V vs. RHE)	$\eta_{cathode}$ (mV)	$Potential_{anode}$ (V vs. RHE)	η_{anode} (mV)
20	10.0	87.8	2.10	-0.47	290	1.56	330
50	5.0	92.7	2.34	-0.54	360	1.70	470
70	5.0	93.3	2.50	-0.60	420	1.77	540
100	5.0	93.6	2.64	-0.70	520	1.82	590
120	5.0	94.0	2.72	-0.73	550	1.84	610
150	4.0	95.0	2.81	-0.75	570	1.87	640
200	4.0	93.7	3.01	-0.76	580	1.90	670
250	13.0	83.0	3.24	-0.80	620	1.94	710

Supplementary Table 11. Various polarization losses in H_2-integrated and conventional CO_2RR process at 50 mA cm^{-2}.

Overpotential (V)	H_2-integrated CO_2RR to CO	H_2-integrated CO_2RR to formate	Water electrolysis	Conventional CO_2RR to CO	Conventional CO_2RR to formate
Ohmic loss	0.11	0.11	-	0.1	0.1
η_{co2RR}	0.42	0.36	-	0.42	0.36
η_{AE1}	0.18	0.18	-	-	-
η_{AE2}	0.03	0.03	-	-	-
η_{OER}	-	-	-	0.47	0.47
η_{HOR}	0.03	0.03	-	-	-
η_{SOEC}	-	-	0.02	-	-
η_{AWE}	-	-	0.20	-	-

Supplementary Table 12. The cell voltage comparison of CO_2RR+HOR (Step 1+2), CO_2RR+NiOR (Step 1) with the state-of-the-art CO_2RR processes in the literature.

Method	Main product of CO_2RR	Current density	Cell voltage	Cell configuration	Reference
CO_2RR+HOR (Step 1+2)	CO	50 mA cm^{-2}	0.87 V	membrane-free flow cell	our work
CO_2RR+NiOR (Step 1)	CO	50 mA cm^{-2}	2.15 V	membrane-free flow cell	our work
CO_2RR+OER	CO	50 mA cm^{-2}	2.0 V	zero-gap flow cell	[1]
CO_2RR+OER	CO	50 mA cm^{-2}	2.17 V	zero-gap flow cell	[2]
CO_2RR+OER	CO	50 mA cm^{-2}	2.35 V	zero-gap flow cell	[3]
CO_2RR+OER	CO	50 mA cm^{-2}	2.50 V	zero-gap flow cell	[4]
CO_2RR+OER	CO	50 mA cm^{-2}	2.62 V	zero-gap flow cell	[5]
CO_2RR+OER	CO	50 mA cm^{-2}	4.27	flow cell	[1]
CO_2RR+GOR	CO	50 mA cm^{-2} (j_{co})	1.32 V	flow cell	[6]
CO_2RR+MOR	$HCOO^-$	50 mA cm^{-2}	1.52 V	H-cell	[7]
CO_2RR+GOR	CO	50 mA cm^{-2}	1.64 V	flow cell	[8]
CO_2RR+UOR	CO	50 mA cm^{-2}	2.25 V	flow cell	[9]
CO_2RR+GOR	$HCOO^-$	50 mA cm^{-2}	2.30 V	flow cell	[10]
CO_2RR+COR	CO	50 mA cm^{-2}	2.47 V	flow cell	[11]
CO_2RR+HMFOR	CO	50 mA cm^{-2}	2.64 V	H-cell	[12]
CO_2RR+COR	CO	50 mA cm^{-2}	3.55 V	H-cell	[13]
CO_2RR+MOR	$HCOO^-$	100 mA cm^{-2}	2.06 V	flow cell	[14]
CO_2RR+SOR	$HCOO^-$	20 mA cm^{-2}	2.0 V	H-cell	[15]
CO_2RR+octylamine electrooxidation	$HCOO^-$	20 mA cm^{-2}	2.20 V	H-cell	[16]
CO_2RR+EDTA-Fe^{2+} oxidation	CO	14 mA cm^{-2}	2.50 V	H-cell	[17]

Notes: **1.** UOR is urea oxidation reactions; HMFOR is 5-hydroxymethyl furfural oxidation reactions; COR is chlorine oxidation reaction; MOR is methanol oxidation reaction; GOR is glycerol oxidation reaction; SOR is sulfide oxidation reaction. **2.** Voltage and current data are obtained from the polarization curves in papers where specific values are not offered.

Supplementary Table 13. The cell voltage and efficiency comparison of "H_2-electricity" conversion (Step 2) with the-state-of-art in the literature at 50 mA cm^{-2}.

Method	Cell voltage	Voltage efficiency	Reference
H_2-electricity conversion (Step 2)	1.28 V	95%	Our work
H_2 fuel cell	0.90 V	73%	[18]
H_2 fuel cell	0.90 V	73%	[19]
H_2 fuel cell	0.89 V	72%	[20]
H_2 fuel cell	0.88 V	72%	[21]
H_2 fuel cell	0.87 V	71%	[22]
H_2 fuel cell	0.82 V	67%	[23]
H_2 fuel cell	0.80 V	65%	[24]
H_2 fuel cell	0.77 V	63%	[25]
H_2 fuel cell	0.72 V	59%	[26]

Note: Voltage and current data are obtained from the polarization curves in papers where specific values are not offered.

Supplementary Table 14. The comparison of energy consumption for CO production between H_2-integrated CO_2RR coupled with water electrolysis and conventional CO_2RR in 1 M KOH at different current densities.

	Current density (mA cm^{-2})	20	50	70	100
H_2-integrated CO_2RR+SOEC	Cell voltage (V) - H_2-integrated CO_2RR	0.51	0.87	1.05	1.26
	CO FE (%)	70.6	71.3	73.1	75.6
	Electricity-CO_2RR (GJ per tonne CO)	5.0	8.4	9.9	11.5
	Cell voltage (V) -SOEC	0.930	0.940	0.945	0.951
	Electricity-water electrolysis (GJ per tonne CO)	9.1	9.1	8.9	8.7
	Energy consumption (GJ per tonne CO)	14.1	17.5	18.8	20.2
H_2-integrated CO_2RR+AWE	Cell voltage (V) - H_2-integrated CO_2RR	0.51	0.87	1.05	1.26
	CO FE (%)	70.6	71.3	73.1	75.6
	Electricity (GJ per tonne CO)	5.0	8.4	9.9	11.5
	Cell voltage (V) -AWE	1.39	1.43	1.46	1.48
	Electricity-water electrolysis (GJ per tonne CO)	13.6	13.8	13.8	13.5
	Energy consumption (GJ per tonne CO)	18.6	22.2	23.7	25.0
conventional CO_2RR	Cell voltage (V) -conventional CO_2RR	2.01	2.34	2.50	2.64
	CO FE (%)	65.5	71.3	72.5	74.6
	Energy consumption (GJ per tonne CO)	21.1	22.4	23.8	24.7

Supplementary Table 15. The comparison of energy consumption for formate production between H_2-integrated CO_2RR coupled with water electrolysis and conventional CO_2RR in 1 M KOH at different current densities.

	Current density (mA cm^{-2})	20	50	70	100
H_2-integrated CO_2RR+SOEC	Cell voltage (V)<br>- H_2-integrated CO_2RR	0.59	0.89	1.05	1.25
	Formate FE (%)	87.8	93.8	94.8	94.3
	Electricity-CO_2RR<br>(GJ per tonne formate)	3.2	4.6	5.3	6.4
	Cell voltage (V)<br>-SOEC	0.930	0.940	0.945	0.951
	Electricity-water electrolysis<br>(GJ per tonne formate)	5.1	4.8	4.8	4.9
	Energy consumption<br>(per tonne formate)	8.3	9.4	10.1	11.3
H_2-integrated CO_2RR+AWE	Cell voltage (V)<br>- H_2-integrated CO_2RR	0.59	0.89	1.05	1.25
	Formate FE (%)	87.8	93.8	94.8	94.3
	Electricity<br>(GJ per tonne formate)	3.2	4.6	5.3	6.4
	Cell voltage (V)<br>-AWE	1.39	1.43	1.46	1.48
	Electricity-water electrolysis<br>(GJ per tonne formate)	7.6	7.4	7.4	7.6
	Energy consumption<br>(GJ per tonne formate)	10.8	12.0	12.7	14.0
conventional CO_2RR	Cell voltage (V)<br>-conventional CO_2RR	2.10	2.34	2.50	2.64
	Formate FE (%)	87.8	92.7	93.3	93.6
	Energy consumption<br>(GJ per tonne formate)	11.6	12.2	12.9	13.6

Supplementary Table 16. The comparison of energy consumption between H_2-integrated CO_2RR coupled with water electrolysis and conventional CO_2RR in 1 M KOH when water electrolyzer was operated at the industrially-relevant conditions.

	CO production		Formate production	
H_2-integrated CO_2RR+SOEC	Cell voltage (V) - H_2-integrated CO_2RR	0.87	Cell voltage (V) - H_2-integrated CO_2RR	0.89
	CO FE (%)	71.3	Formate FE (%)	93.8
	Electricity-CO_2RR (GJ per tonne CO)	8.4	Electricity-CO_2RR (GJ per tonne formate)	4.6
	Cell voltage (V) -SOEC	1.28	Cell voltage (V) -SOEC	1.28
	Electricity-water electrolysis (GJ per tonne CO)	12.4	Electricity-water electrolysis (GJ per tonne formate)	6.6
	Energy consumption (GJ per tonne CO)	20.8	Energy consumption (GJ per tonne formate)	11.2
H_2-integrated CO_2RR+AWE	Cell voltage (V) - H_2-integrated CO_2RR	0.87	Cell voltage (V) - H_2-integrated CO_2RR	0.89
	CO FE (%)	71.3	Formate FE (%)	93.8
	Electricity (GJ per tonne CO)	8.4	Electricity (GJ per tonne formate)	4.6
	Cell voltage (V) -AWE	1.47	Cell voltage (V) -AWE	1.47
	Electricity-water electrolysis (GJ per tonne CO)	14.2	Electricity-water electrolysis (GJ per tonne formate)	7.6
	Energy consumption (GJ per tonne CO)	22.6	Energy consumption (GJ per tonne formate)	12.2
conventional CO_2RR	Cell voltage (V) -conventional CO_2RR	2.34	Cell voltage (V) -conventional CO_2RR	2.34
	CO FE (%)	71.3	Formate FE (%)	92.7
	Energy consumption (GJ per tonne CO)	22.4	Energy consumption (GJ per tonne formate)	12.2

Supplementary Table 17. The comparison of energy consumption for CO production between H_2-integrated CO_2RR coupled with water electrolysis and conventional CO_2RR in 1 M $KHCO_3$ at different current densities.

	Current density (mA cm^{-2})	20	50	70	100
H_2-integrated CO_2RR+SOEC	Cell voltage (V) H_2-integrated CO_2RR	0.93	1.21	1.38	1.59
	CO FE (%)	60.6	63.3	66.1	70.6
	Electricity-CO_2RR (GJ per tonne CO)	10.6	13.2	14.4	15.5
	Anode gas separation (GJ per tonne CO)	0	0	0	0
	Cell voltage (V) -SOEC	0.930	0.940	0.945	0.951
	Electricity-water electrolysis (GJ per tonne CO)	10.6	10.2	9.9	9.3
	Energy consumption (GJ per tonne CO)	21.2	23.4	24.3	24.8
H_2-integrated CO_2RR+AWE	Cell voltage (V) - H_2-integrated CO_2RR	0.93	1.21	1.38	1.59
	CO FE (%)	60.6	63.3	66.1	70.6
	Electricity (GJ per tonne CO)	10.6	13.2	14.4	15.5
	Anode gas separation (GJ per tonne CO)	0	0	0	0
	Cell voltage (V) -AWE	1.39	1.43	1.46	1.48
	Electricity-water electrolysis (GJ per tonne CO)	15.8	15.6	15.2	14.4
	Energy consumption (GJ per tonne CO)	26.4	28.8	29.6	29.9
conventional CO_2RR	Cell voltage (V) -conventional CO_2RR	2.46	2.66	2.84	3.01
	CO FE (%)	55.8	62.8	65.5	70.6
	Electricity (GJ per tonne CO)	30.4	29.2	29.9	29.4
	Anode gas separation (GJ per tonne CO)	11.3	10.0	9.6	8.9
	Energy consumption (GJ per tonne CO)	41.7	39.2	39.5	38.3

Note: At 100 mA cm^{-2}, the geometric area of mediator was 3×3 cm^2.

Supplementary Table 18. The comparison of energy consumption for formate production between H_2-integrated CO_2RR coupled with water electrolysis and conventional CO_2RR in 1 M $KHCO_3$ at different current densities.

	Current density (mA cm^{-2})	20	50	70	100
H_2-integrated CO_2RR+SOEC	Cell voltage (V) - H_2-integrated CO_2RR	0.84	1.2	1.44	1.79
	Formate FE (%)	81.8	85.8	89.8	89.3
	Electricity-CO_2RR (GJ per tonne formate)	5.0	6.7	7.7	9.7
	Anode gas separation (GJ per tonne formate)	0	0	0	0
	Cell voltage (V) -SOEC	0.930	0.940	0.945	0.951
	Electricity-water electrolysis (GJ per tonne formate)	5.5	5.3	5.1	5.1
	Energy consumption (GJ per tonne formate)	10.5	12.0	12.8	14.8
H_2-integrated CO_2RR+AWE	Cell voltage (V) - H_2-integrated CO_2RR	0.84	1.2	1.44	1.79
	Formate FE (%)	81.8	85.8	89.8	89.3
	Electricity (GJ per tonne formate)	5.0	6.7	7.7	9.7
	Anode gas separation (GJ per tonne formate)	0	0	0	0
	Cell voltage (V) -AWE	1.39	1.43	1.46	1.48
	Electricity-water electrolysis (GJ per tonne formate)	8.2	8.0	7.8	8.0
	Energy consumption (GJ per tonne formate)	13.2	14.7	15.5	17.7
conventional CO_2RR	Cell voltage (V) -conventional CO_2RR	2.36	2.7	2.9	3.2
	Formate FE (%)	81.8	84.7	88.8	89.3
	Electricity (GJ per tonne formate)	13.9	15.4	15.8	17.3
	Anode gas separation (GJ per tonne formate O)	5.4	5.2	5.0	4.9
	Energy consumption (GJ per tonne formate)	19.3	20.6	20.8	22.2

Note: At 100 mA cm^{-2}, the geometric area of mediator was 3×3 cm^2.

Supplementary Table 19. The comparison of energy consumption between H_2-integrated CO_2RR coupled with water electrolysis and conventional CO_2RR in 1 M $KHCO_3$ when water electrolyzer was operated at the industrially-relevant conditions.

	CO production		Formate production	
H_2-integrated CO_2RR+SOEC	Cell voltage (V)<br>- H_2-integrated CO_2RR	1.21	Cell voltage (V)<br>- H_2-integrated CO_2RR	1.2
	CO FE (%)	63.3	Formate FE (%)	85.8
	Electricity-CO_2RR<br>(GJ per tonne CO)	13.2	Electricity-CO_2RR<br>(GJ per tonne formate)	6.7
	Anode gas separation<br>(GJ per tonne CO)	0	Anode gas separation<br>(GJ per tonne formate)	0
	Cell voltage (V)<br>-SOEC	1.28	Cell voltage (V)<br>-SOEC	1.28
	Electricity-water electrolysis<br>(GJ per tonne CO)	13.9	Electricity-water electrolysis<br>(GJ per tonne formate)	7.2
	Energy consumption<br>(GJ per tonne CO)	27.1	Energy consumption<br>(GJ per tonne formate)	13.9
H_2-integrated CO_2RR+AWE	Cell voltage (V)<br>- H_2-integrated CO_2RR	1.21	Cell voltage (V)<br>- H_2-integrated CO_2RR	1.2
	CO FE (%)	63.3	Formate FE (%)	85.8
	Electricity<br>(GJ per tonne CO)	13.2	Electricity<br>(GJ per tonne formate)	6.7
	Anode gas separation<br>(GJ per tonne CO)	0	Anode gas separation<br>(GJ per tonne formate)	0
	Cell voltage (V)<br>-AWE	1.47	Cell voltage (V)<br>-AWE	1.47
	Electricity-water electrolysis<br>(GJ per tonne CO)	16.0	Electricity-water electrolysis<br>(GJ per tonne formate)	8.3
	Energy consumption<br>(GJ per tonne CO)	29.2	Energy consumption<br>(GJ per tonne formate)	15.0
conventional CO_2RR	Cell voltage (V)<br>-conventional CO_2RR	2.66	Cell voltage (V)<br>-conventional CO_2RR	2.70
	CO FE (%)	62.8	Formate FE (%)	84.7
	Electricity<br>(GJ per tonne CO)	29.2	Electricity<br>(GJ per tonne formate)	15.4
	Anode gas separation<br>(GJ per tonne CO)	10.0	Anode gas separation<br>(GJ per tonne formate)	5.2
	Energy consumption<br>(GJ per tonne CO)	39.2	Energy consumption<br>(GJ per tonne formate)	20.6

Supplementary Table 20. The unit cost of component materials.

Component	Cost	Units	Source
Zn-Cu foam GDE	12.8	\$ m^{-2}	a
Co_3O_4 electrode	9.8	\$ m^{-2}	a
$Ni(OH)_2$/NiOOH	14.0	\$ m^{-2}	a
Pt GDE	16.0	\$ m^{-2}	a
Separator	2.0	\$ m^{-2}	[27]
Nafion membrane	350	\$ m^{-2}	[28]
Cathode of SOEC (Ni+YSZ)	81.5	\$ m^{-2}	a
Anode of SOEC (LSM)	53.8	\$ m^{-2}	a

a. based on the chemicals/materials cost in Supplementary Table 21.

Supplementary Table 21. The cost of chemicals and materials.

Chemicals/materials	Cost	Units	Source
Carbon black	0.04	$ kg^{-1}	a
PTFE	10.3	$ kg^{-1}	b
Zn	8.0	$ kg^{-1}	c
Cu foam	2.4	$ m^{-2}	c
Co	75.0	$ kg^{-1}	c
Ni foam	6.0	$ m^{-2}	[29]
Ni	14.0	$ kg^{-1}	c
Pt	32035.0	$ kg^{-1}	c
NiO	14.0	$ kg^{-1}	c
ZrO_2	45.8	$ kg^{-1}	d
$La_{0.8}Sr_{0.2}MnO_{3-x}$	7000.0	$ kg^{-1}	e
GDL	0.006	$ m^{-2}	[30]

a. Taken from online report of "carbon black price trend and forecast" (https://www.chemanalyst.com/).

b. Taken from online report of "PTFE prices rising due to improvement in downstream market demand" (https://www.chemanalyst.com/).

c. Taken from the report of daily metal prices (https://www.dailymetalprice.com/).

d. Taken from online trade market. (https://www.made-in-china.com/products-search/hot-china-products/Zro2_Zirconia_Powder_Price.html)

e. Taken from online trade market. (https://www.sigmaaldrich.cn/CN/zh/product/aldrich/704261)

2. Supplementary Figures

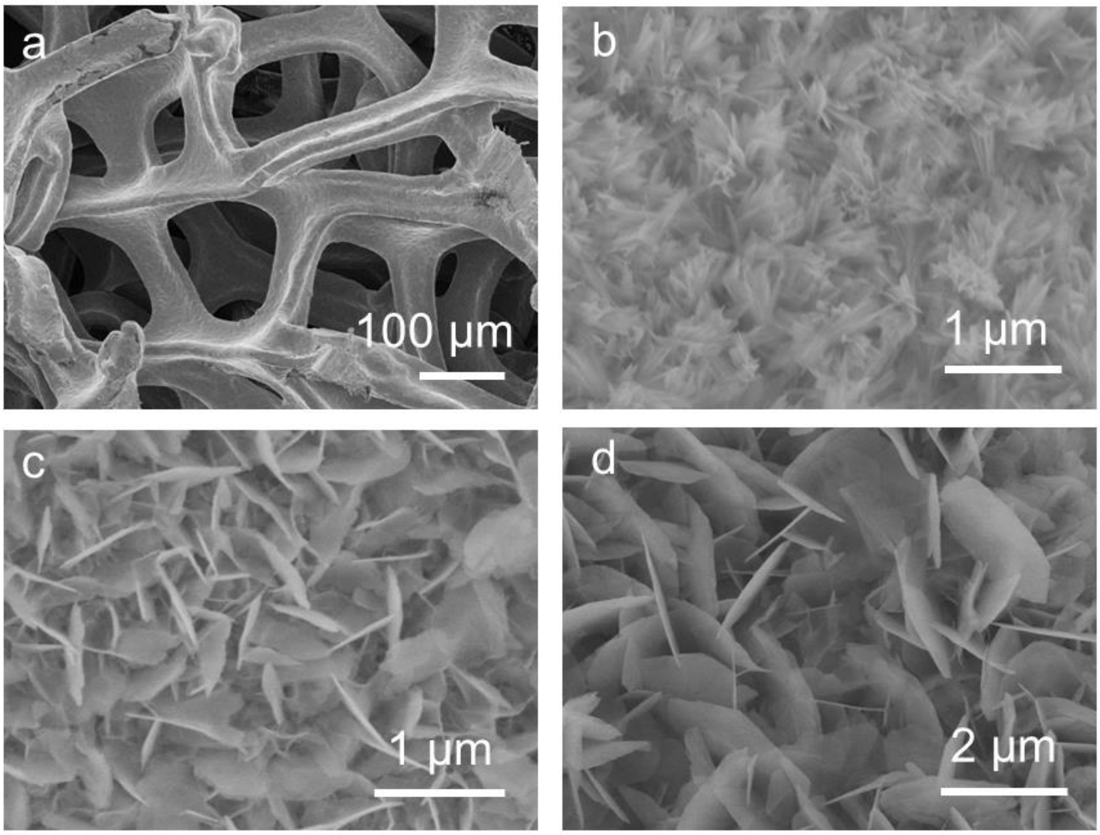

Supplementary Fig. 1. The surface morphology of Zn-Cu electrode. SEM images of (a) Cu foam, (b) Zn-Cu-100, (c) Zn-Cu-500, and (d) Zn-Cu-1000.

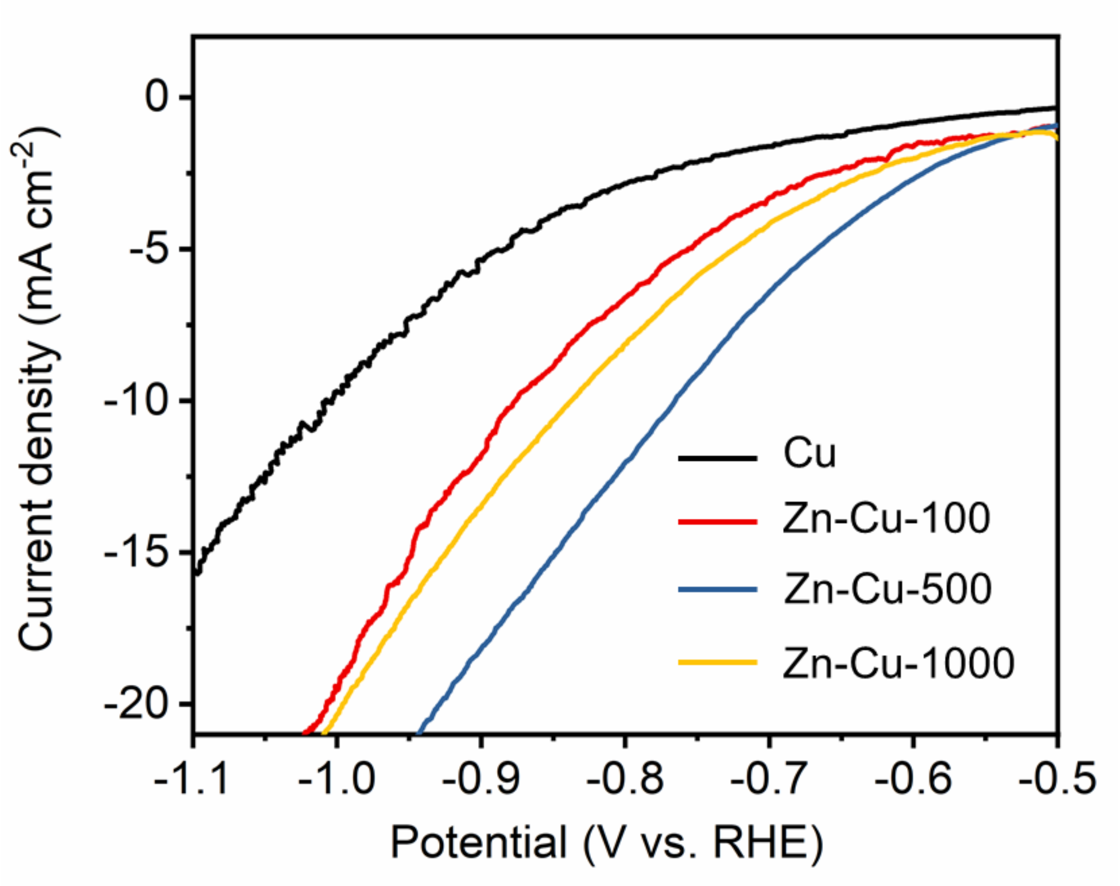

Supplementary Fig. 2. The electrochemical performance of Zn-Cu electrode. LSV curves of Cu, Zn-Cu-100, Zn-Cu-500, and Zn-Cu-1000 in CO_2-saturated 0.1 M $KHCO_3$ electrolyte with a scan rate of 5 mV s^{-1}. The solution resistance of Cu, Zn-Cu-100, Zn-Cu-500, and Zn-Cu-1000 electrodes were 7.89, 8.18, 8.83, and 7.75 Ω, respectively.

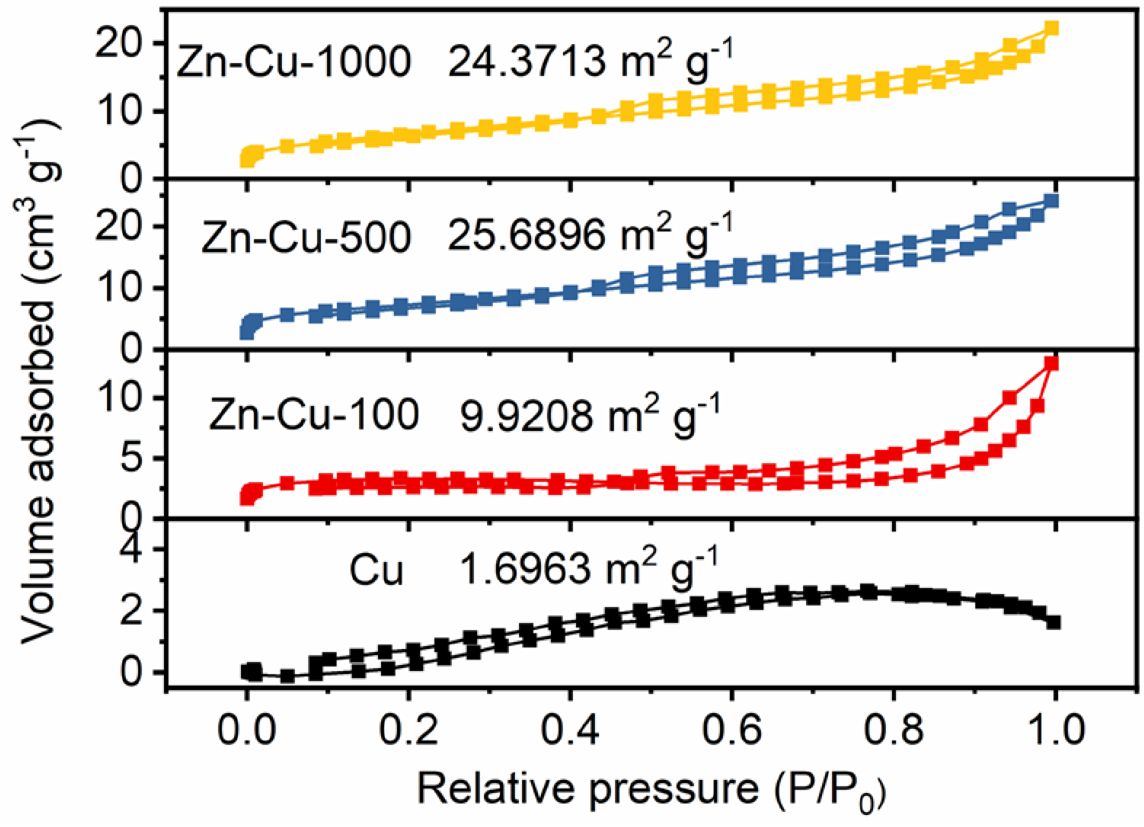

Supplementary Fig. 3. The specific surface area of Zn electrode. Nitrogen adsorption-desorption isotherms of Cu, Zn-Cu-100, Zn-Cu-500, and Zn-Cu-1000.

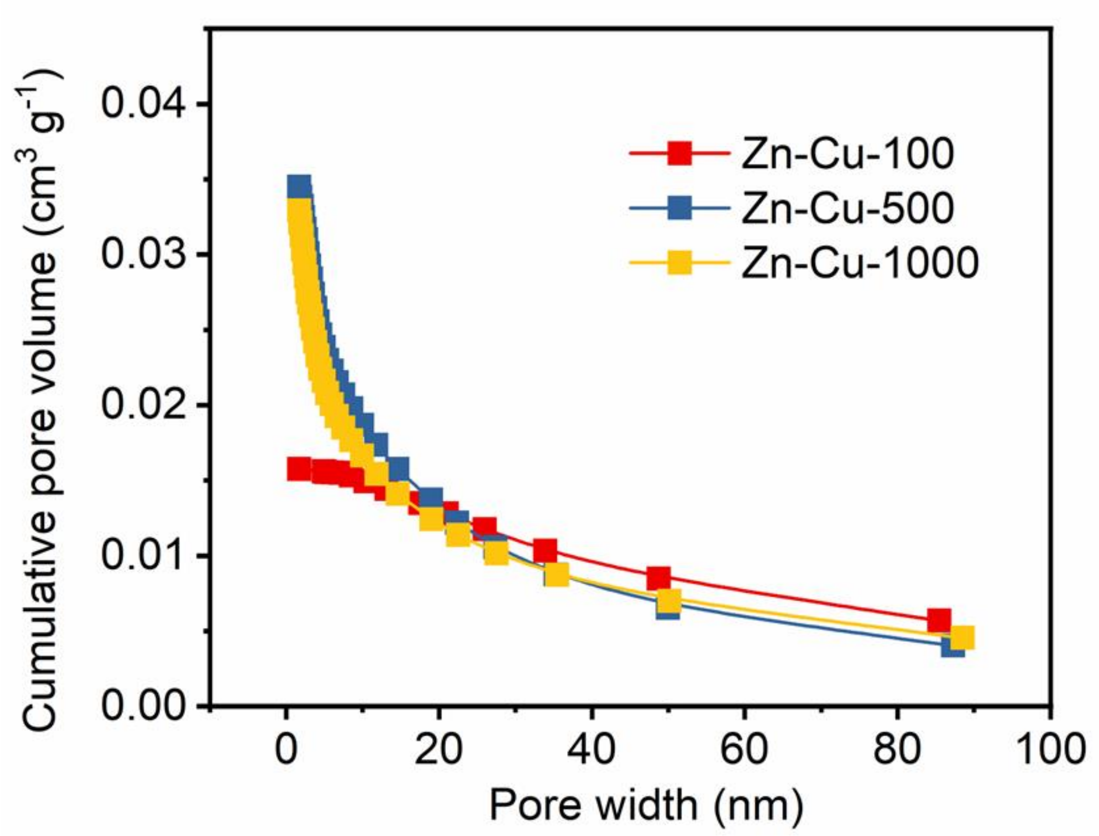

Supplementary Fig. 4. Cumulative pore volume distributions of Zn-Cu-100, Zn-Cu-500, and Zn-Cu-1000.

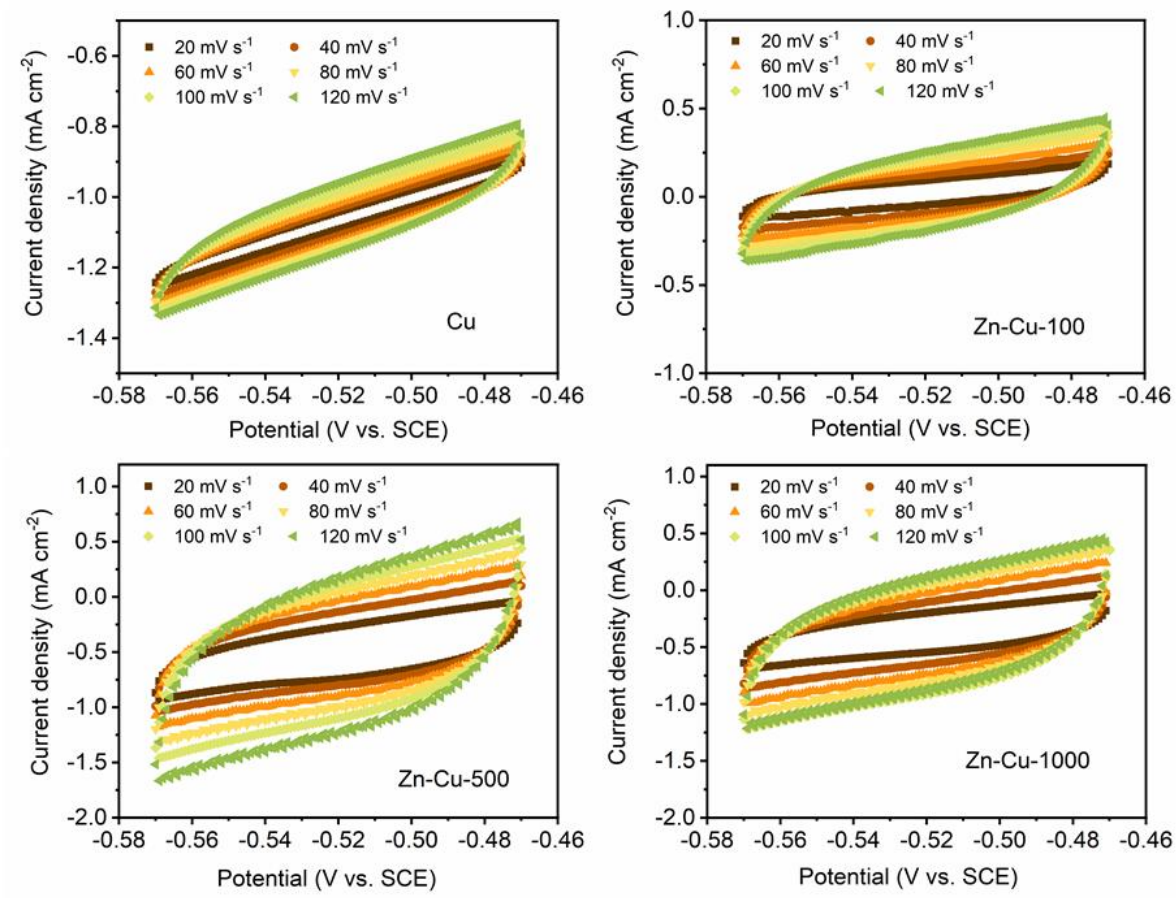

Supplementary Fig. 5. Capacitive behaviors of Zn electrodes. CV curves of Cu, Zn-Cu-100, Zn-Cu-500, and Zn-Cu-1000 with a potential range from -0.46 to -0.57 V vs. SCE in an Ar-saturated 0.5 M Na_2SO_4 electrolyte.

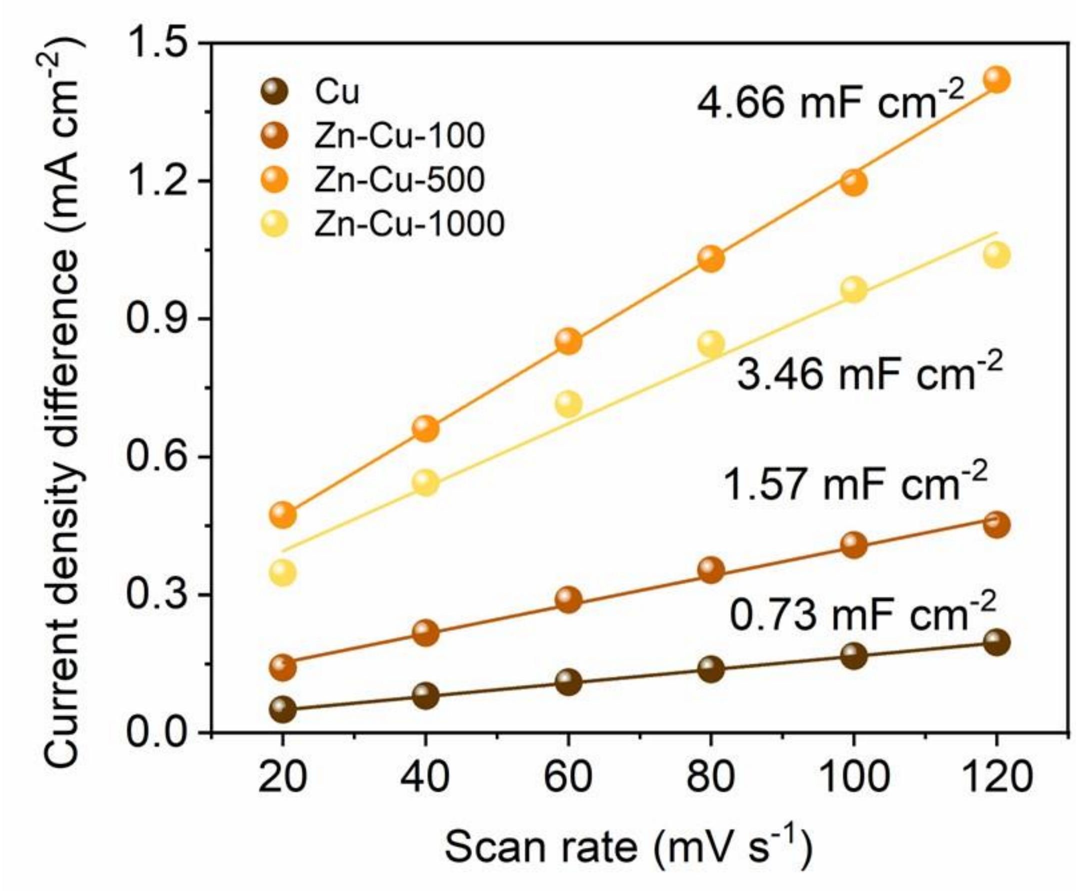

Supplementary Fig. 6. The capacitance (C_{dl}) of Zn electrodes. The plot of charging current density difference versus the scan rate.

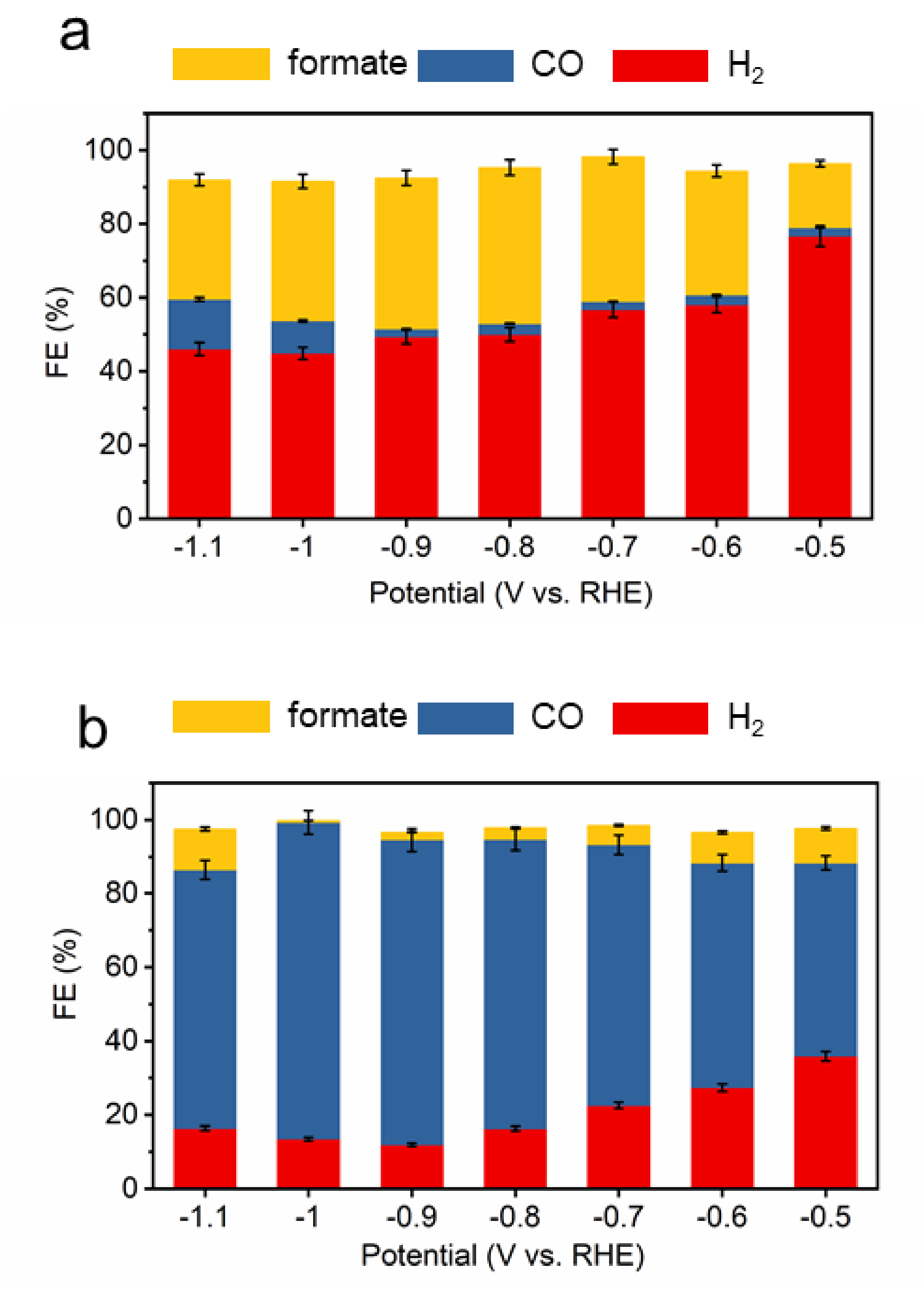

Supplementary Fig. 7. The product distribution of CO_2RR on Zn electrode. FE of various products on (a) Cu and (b) Zn-Cu-500 electrode at different potentials in a H-cell. The error bar represents standard deviation from three independent measurements.

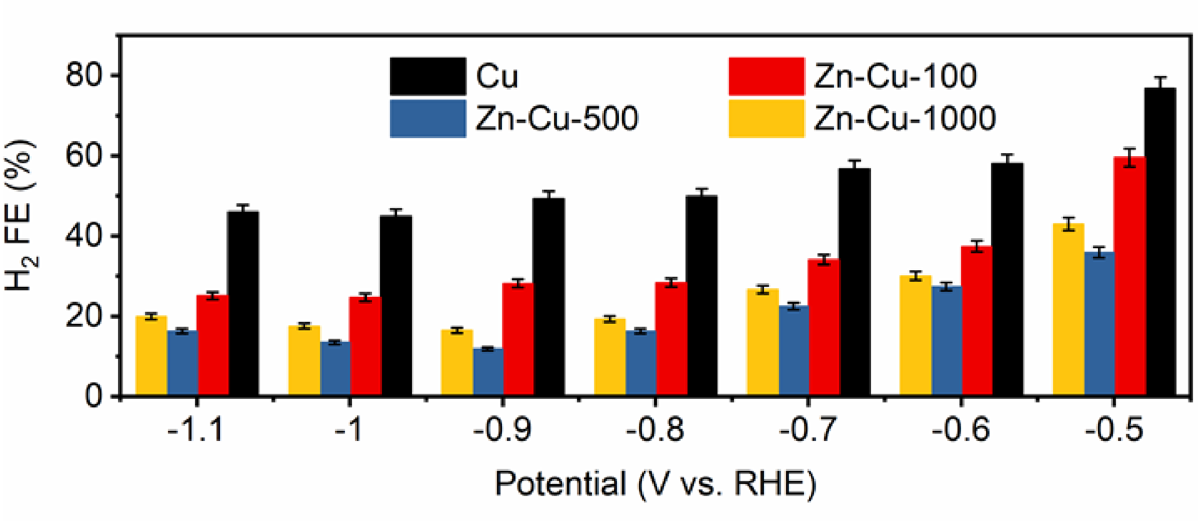

Supplementary Fig. 8. H_2 FE of Cu, Zn-Cu-100, Zn-Cu-500 and Zn-Cu-1000 at different potentials in a H-cell. The error bar represents standard deviation from three independent measurements.

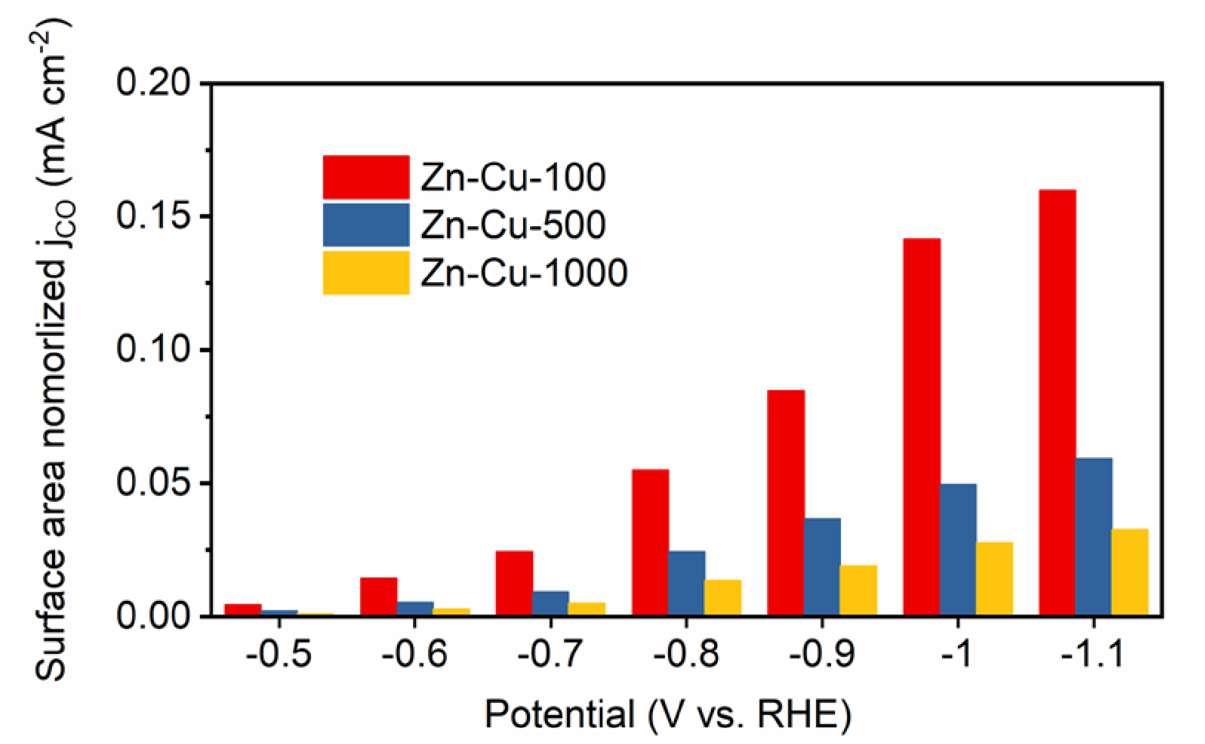

Supplementary Fig. 9. The surface-area-normalized current densities of Zn-Cu-100, Zn-Cu-500 and Zn-Cu-1000 at different potentials.

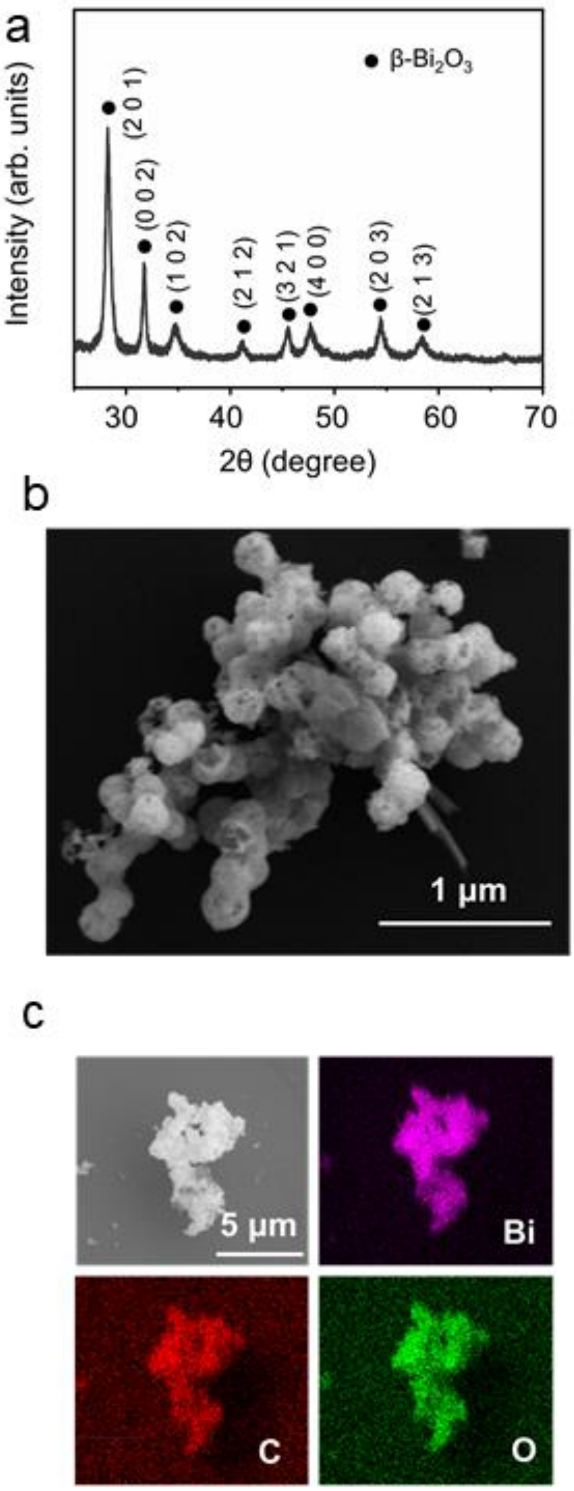

Supplementary Fig. 10. Characterizations of Bi_2O_3 electrode. (a) XRD pattern, (b) SEM image and (c) EDS elemental mappings of nanosphere Bi_2O_3.

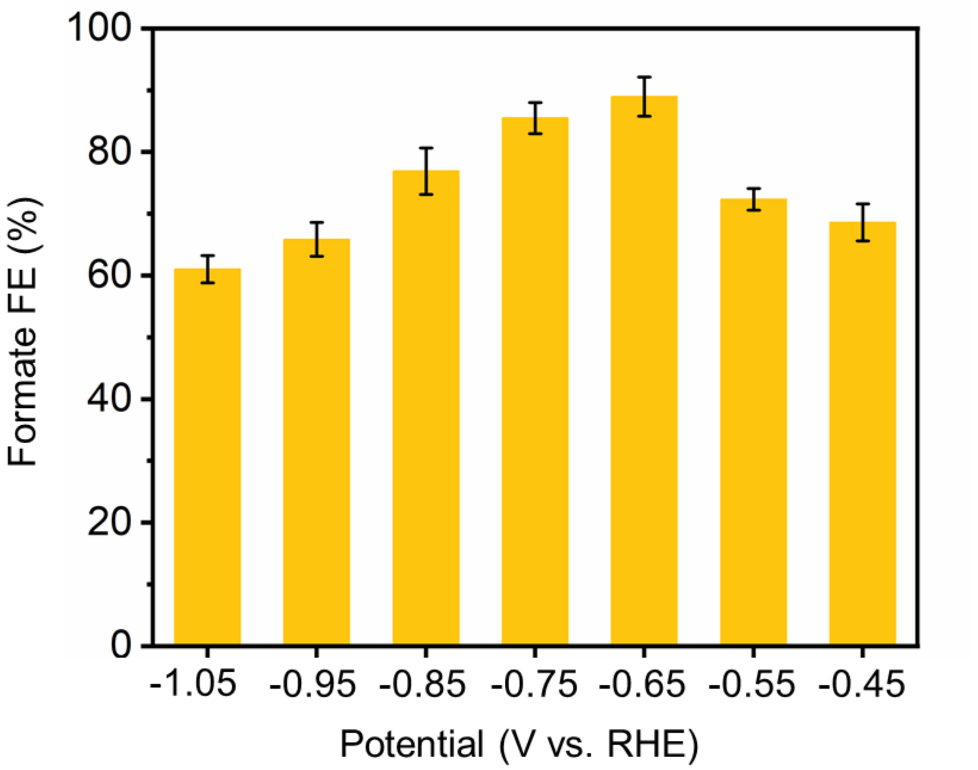

Supplementary Fig. 11. Formate FE of Bi_2O_3 at different potentials in a H-cell. The error bar represents standard deviation from three independent measurements.

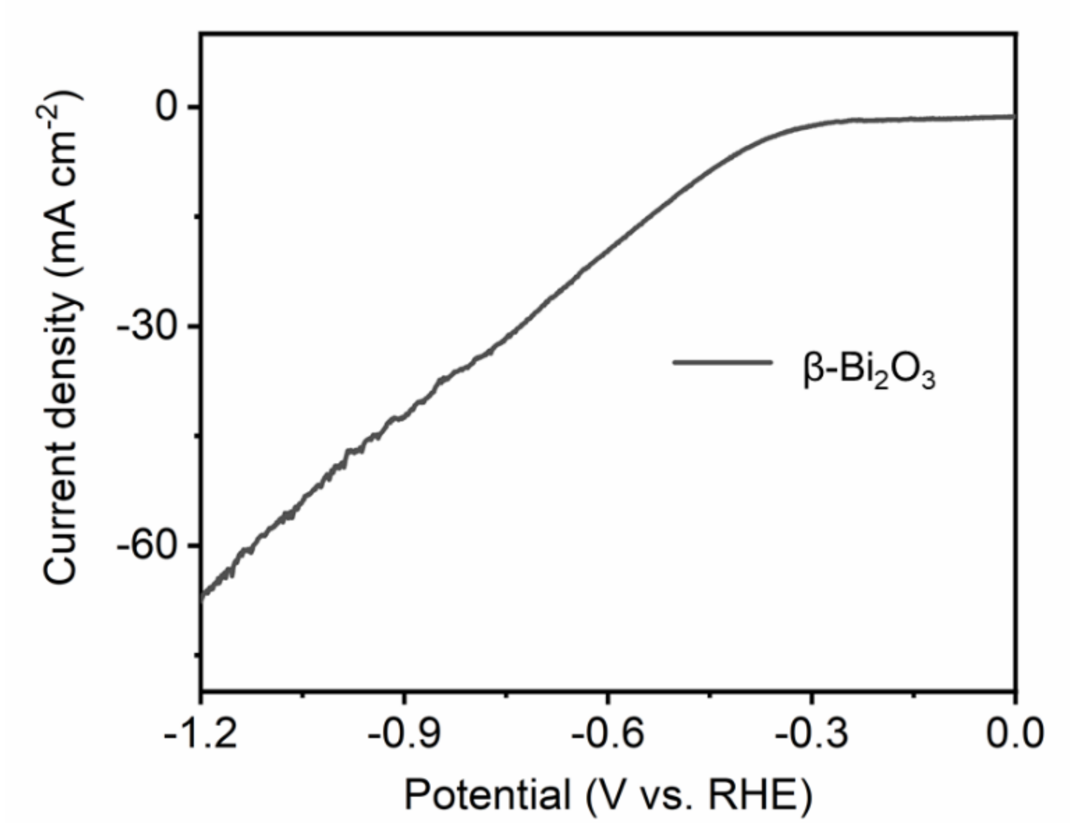

Supplementary Fig. 12. The electrochemical performance of Bi_2O_3 electrode. LSV curve of Bi_2O_3 in CO_2-saturated 0.5 M $KHCO_3$ electrolyte with a scan rate of 5 mV s^{-1}.

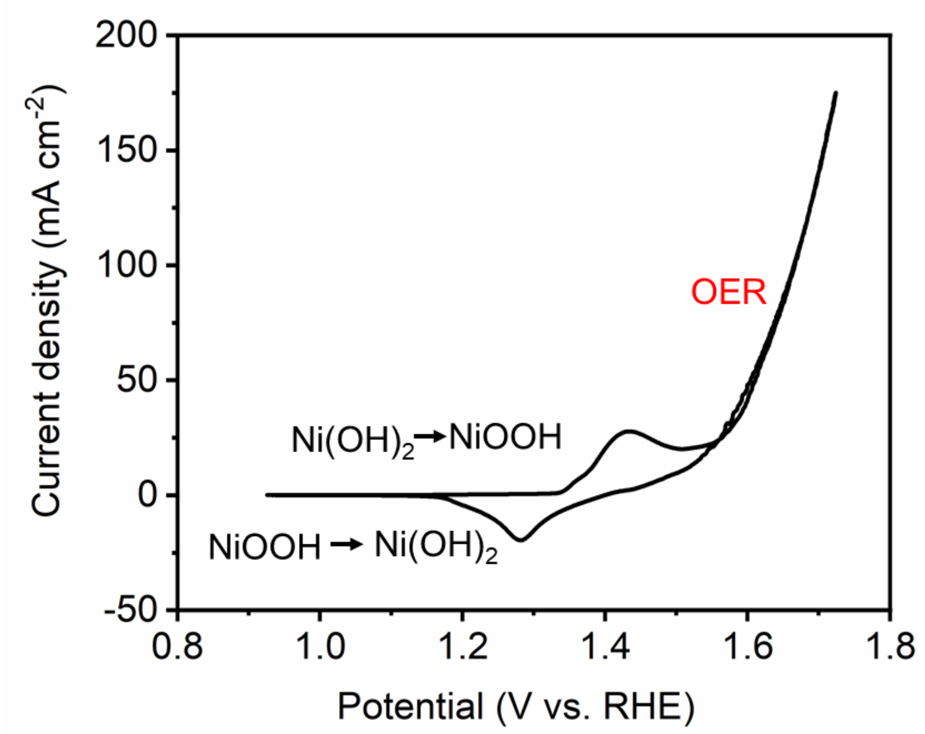

Supplementary Fig. 13. The electrochemical performance of $Ni(OH)_2$ electrode. CV of the $Ni(OH)_2$ electrode at scan rate of 1 mV s^{-1} in 1.0 M KOH at 25 °C. The solution resistance was 1.35 Ω.

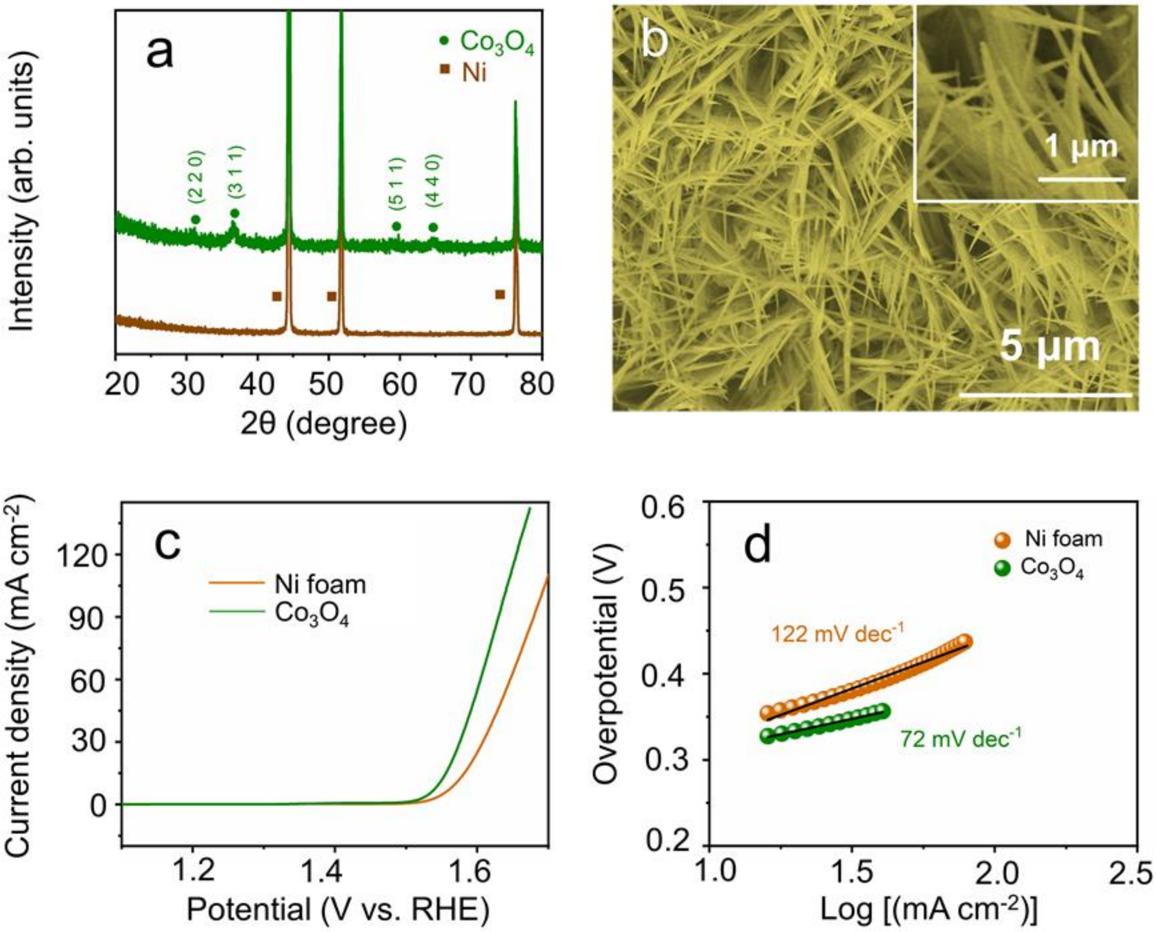

Supplementary Fig. 14. Characterizations and performances of Co_3O_4 electrodes. (a) XRD patterns of Ni foam and Co_3O_4-Ni; (b) SEM image of Co_3O_4 nanowires on the Ni foam; (c) LSV results of Ni foam and Co_3O_4-Ni; (d) Tafel plots of OER on Co_3O_4-Ni and Ni foam. The solution resistances of Ni foam and Co_3O_4 electrode were 1.07 and 1.25 Ω, respectively.

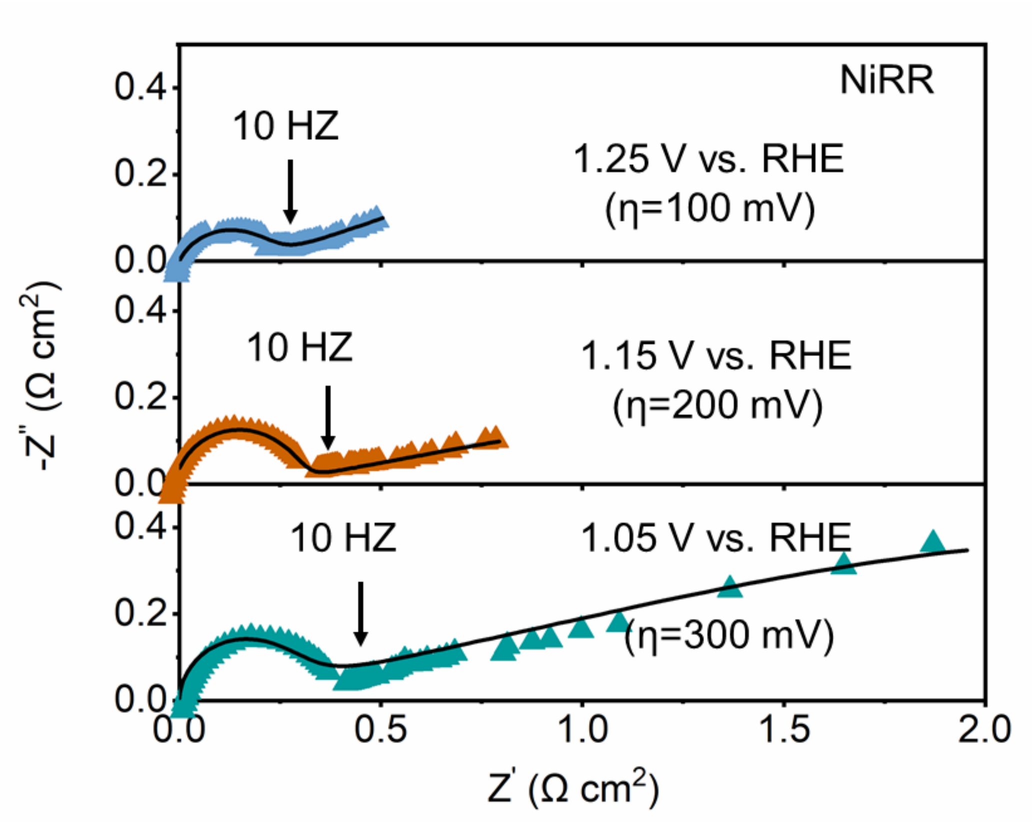

Supplementary Fig. 15. Nyquist plots of $Ni(OH)_2$/NiOOH electrode acquired at 1.25, 1.15, and 1.05 V vs. RHE.

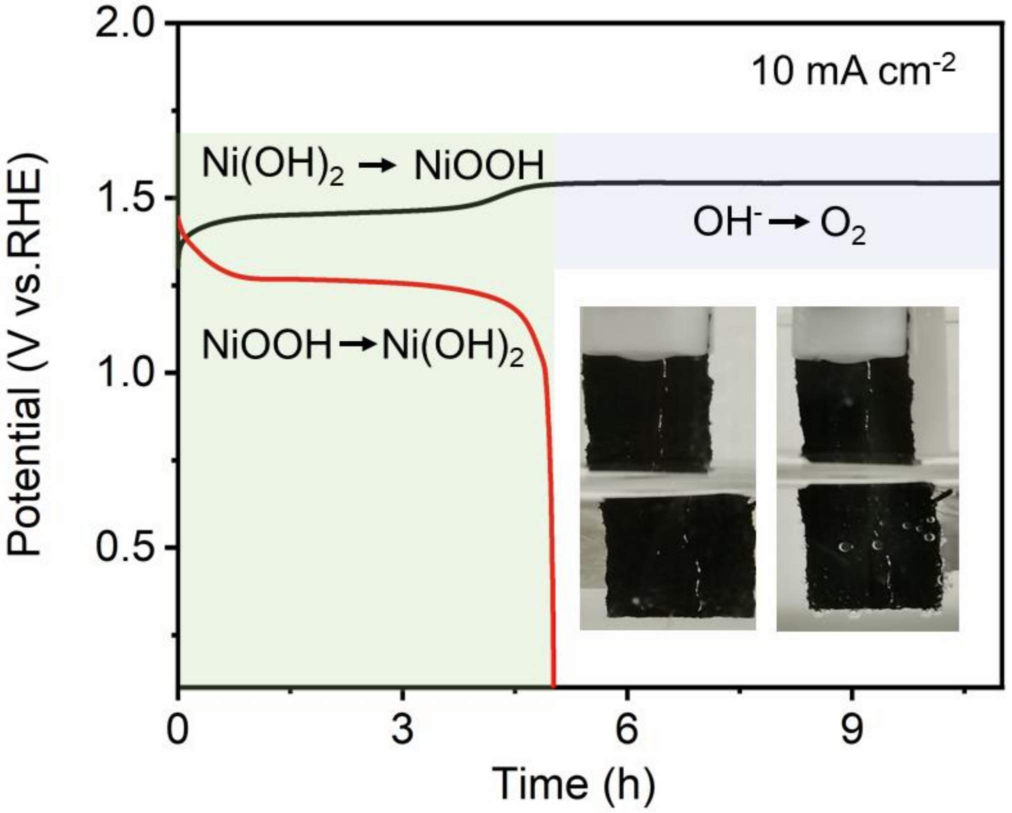

Supplementary Fig. 16. Chronopotentiometric curve of $Ni(OH)_2$/NiOOH conversion at a constant current of 10 mA cm^{-2}; inset shows photo profiles of $Ni(OH)_2$ oxidation (left) and overoxidation of $Ni(OH)_2$ (right) which leads to OER with the bubble evolution.

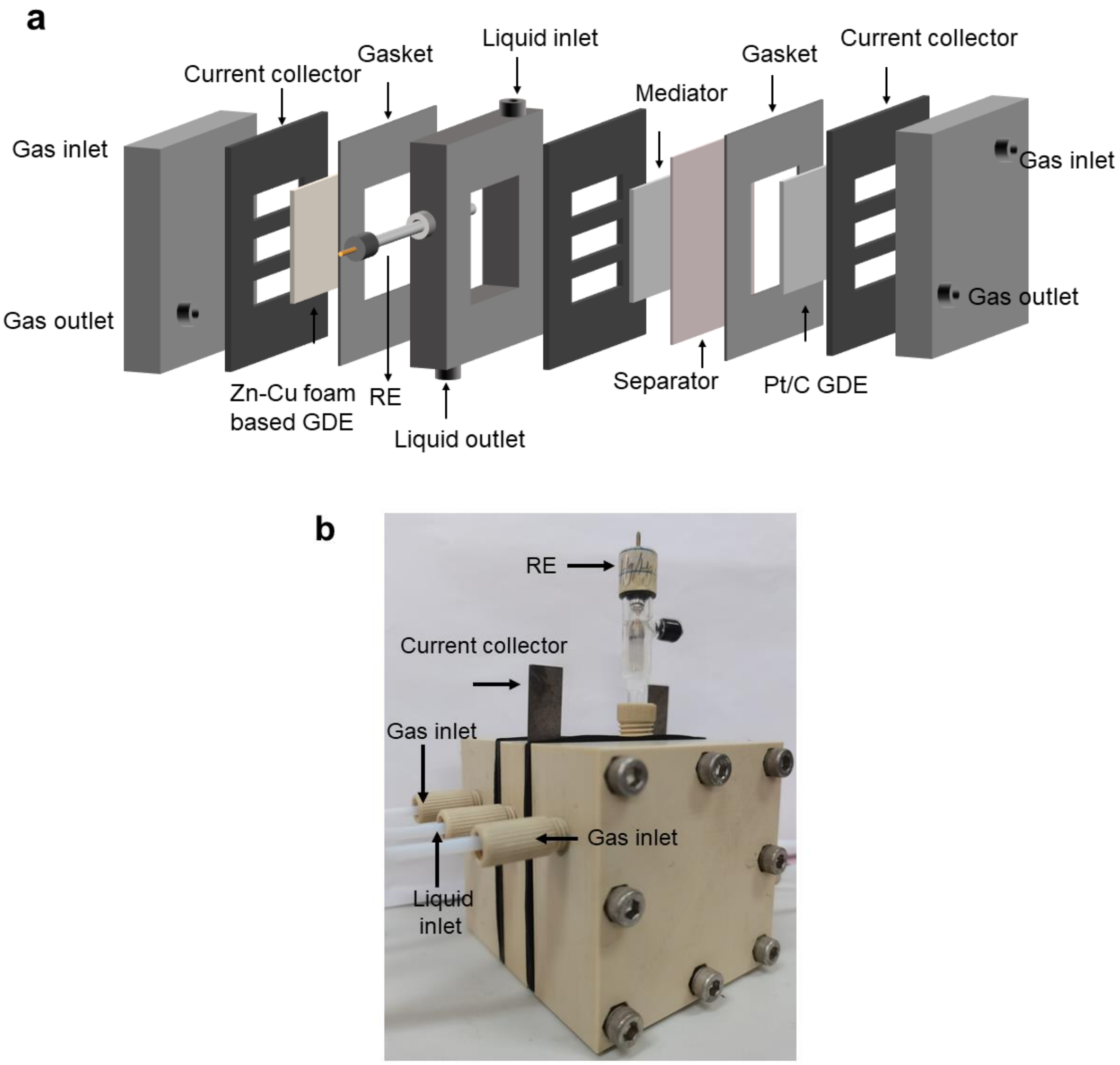

Supplementary Fig. 17. The cell structure of H_2-integrated CO_2RR. (a) schematic representation of the cell assembly; (b) the photograph of the cell.

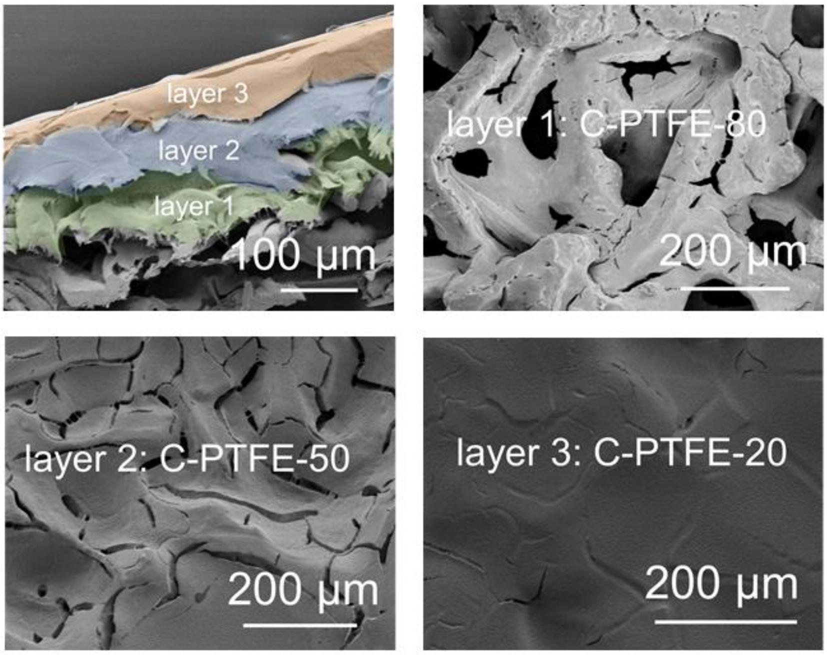

Supplementary Fig. 18. The morphology of Zn-Cu-500 foam GDE. SEM images of the gradient functional layer fabricated via the "layer-by-layer" method and top views of the surface of each layer.

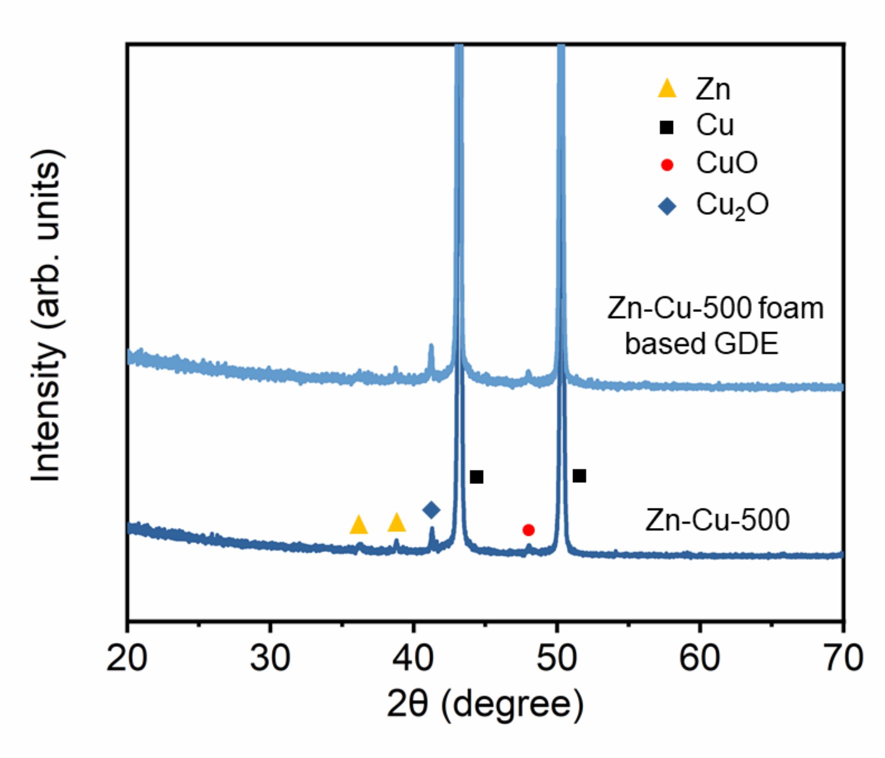

Supplementary Fig. 19. XRD patterns of Zn-Cu-500 foam and Zn-Cu-500 foam based GDE.

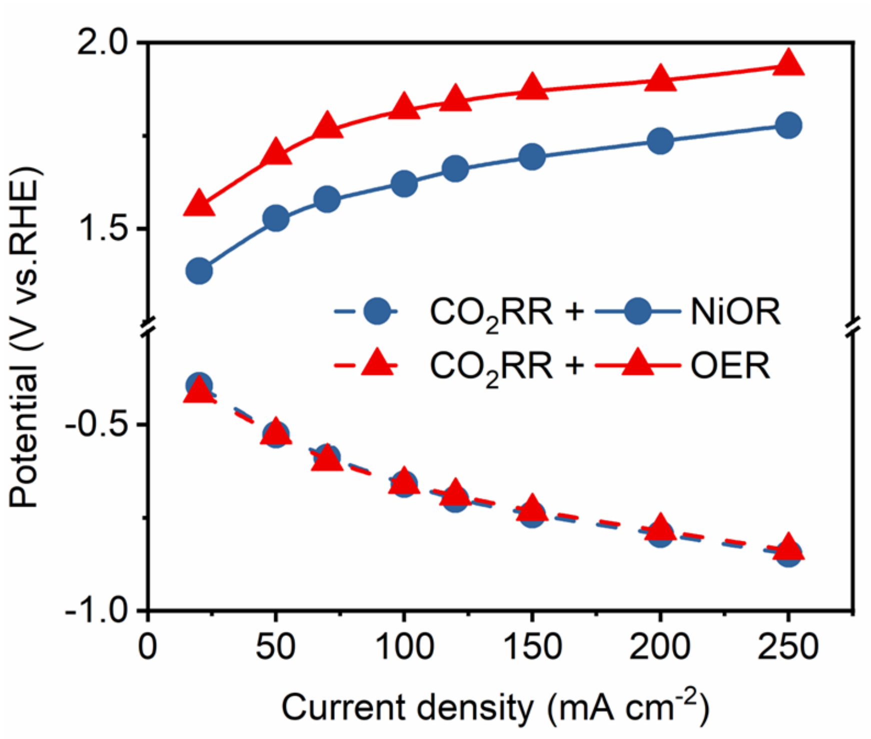

Supplementary Fig. 20. Polarization curves of cathode and anode in the combined CO_2RR+OER and CO_2RR+NiOR processes for CO production.

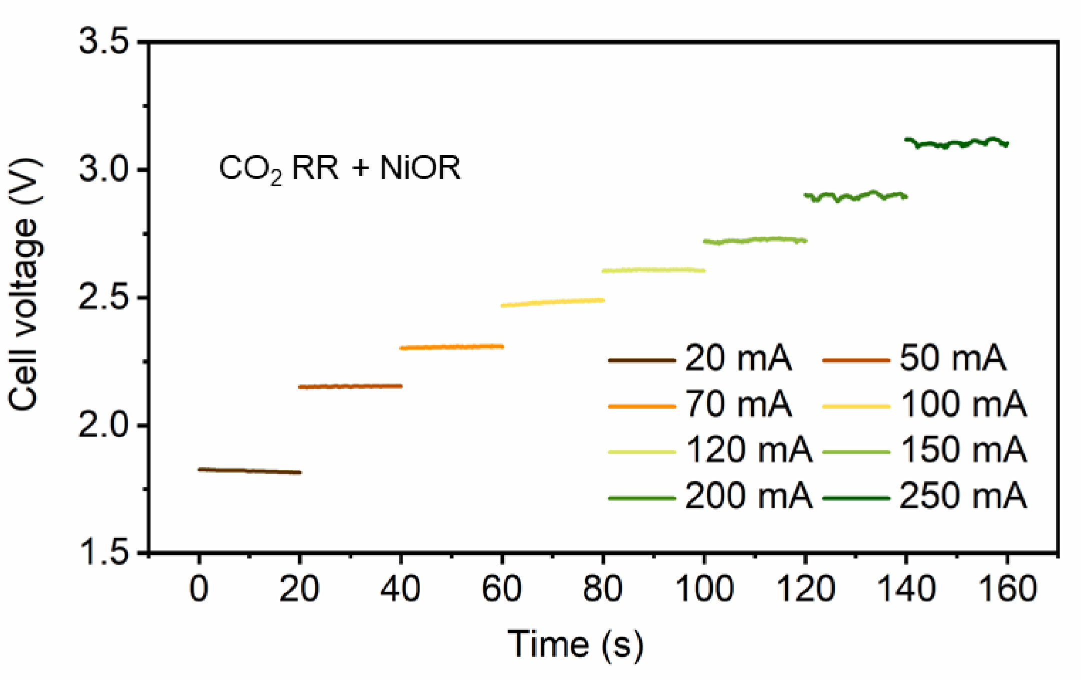

Supplementary Fig. 21. Representative potentials to obtain the polarization curve of CO_2RR+NiOR at 20, 50, 70, 100, 120, 150, 200, and 250 mA cm^{-2} for CO production.

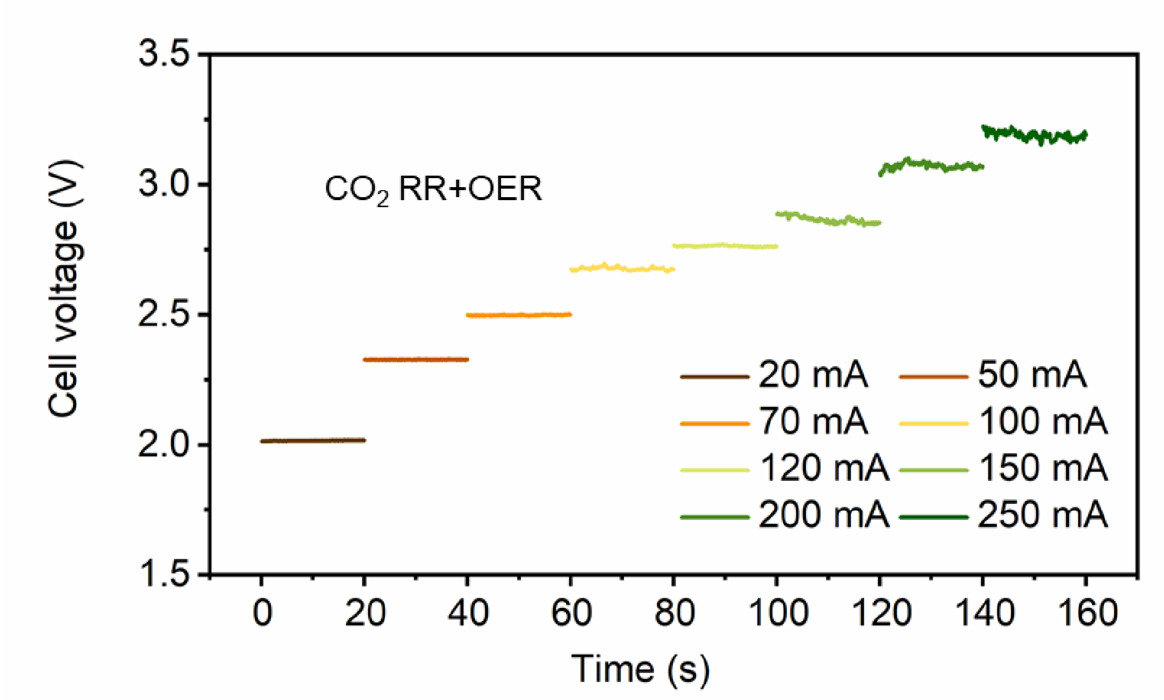

Supplementary Fig. 22. Representative potentials to obtain the polarization curve of CO_2RR+OER at 20, 50, 70, 100, 120, 150, 200, and 250 mA cm^{-2} for CO production.

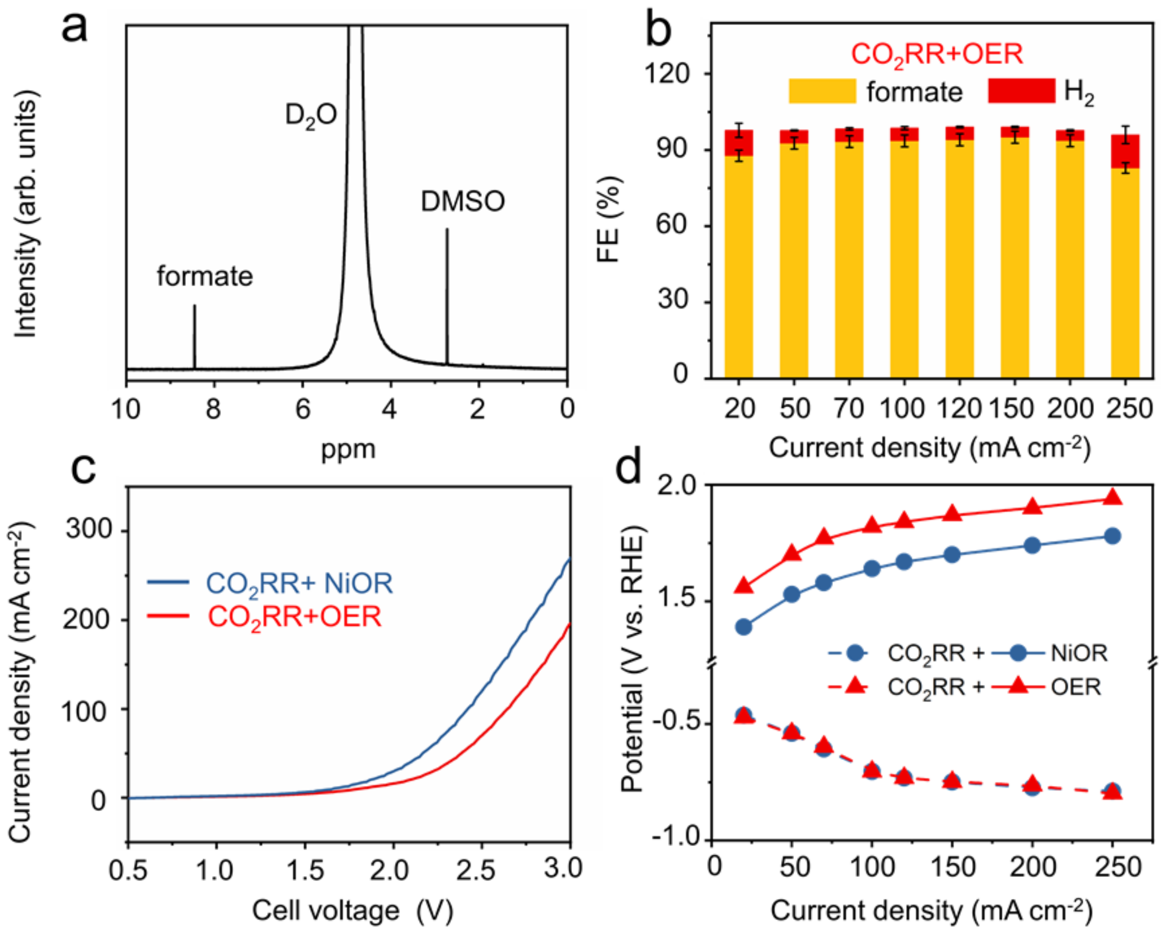

Supplementary Fig. 23. CO_2RR performance for formate production in the cell. (a) ^{1}H NMR spectra of the cathode effluent from the cell based on a Bi_2O_3 GDE; (b) Faradaic efficiencies in CO_2RR+OER system for formate production (the error bar represents standard deviation from three independent measurements); (c) polarization curves of CO_2RR+NiOR and CO_2RR+OER for formate production; (d) polarization curves of cathode and anode in the combined CO_2RR+OER and CO_2RR+NiOR processes for formate production.

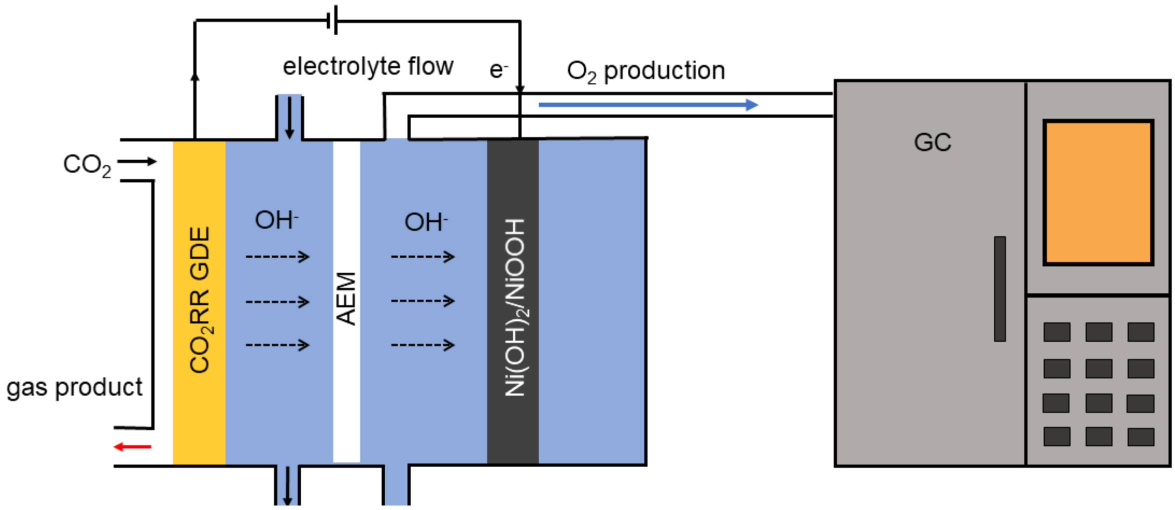

Supplementary Fig. 24. The schematic illustration of the cell optimization to measure O_2 production at the anode side. The anode and cathode compartments were separated by a 130 μm-thick anion-exchange membrane (AEM, Fumasep FAA-3-PK-130). Before test, AEM was immersed in a 0.1 M KOH solution for 6 h.

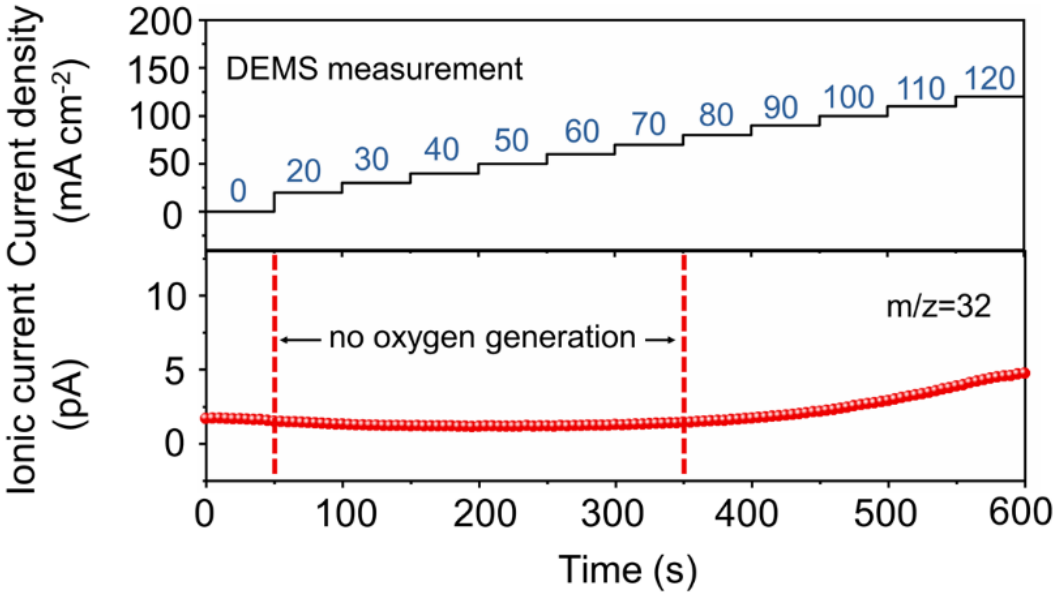

Supplementary Fig. 25. Monitoring O_2 production by DEMS. The ionic current response in the DEMS measurement in which $Ni(OH)_2$ was used as the working electrode biased at various constant current densities.

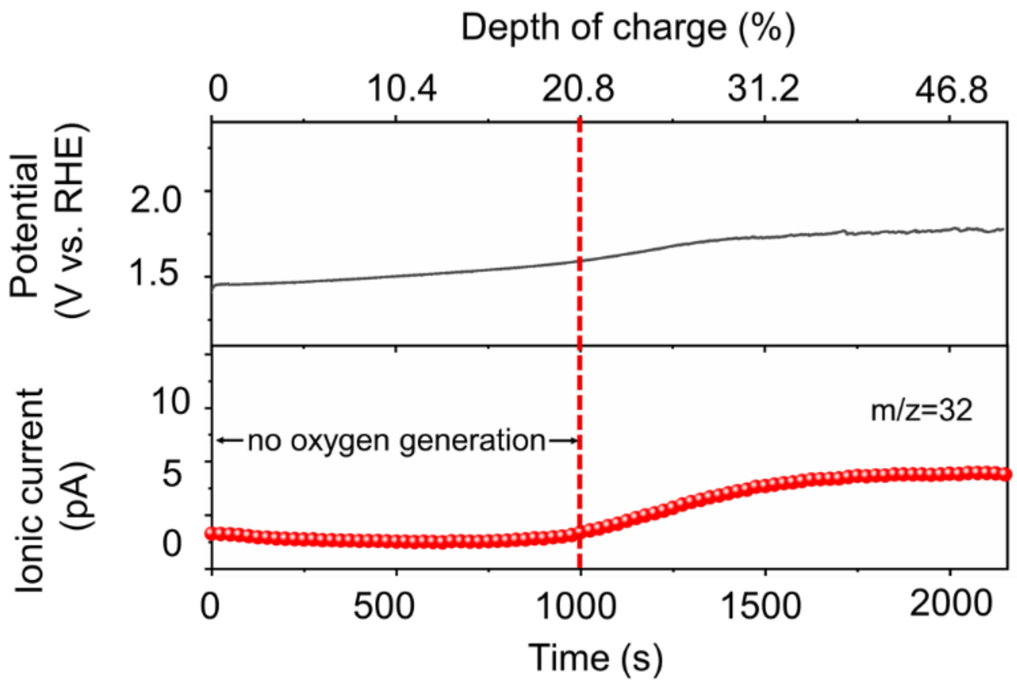

Supplementary Fig. 26. Monitoring O_2 production by DEMS. The ionic current response in the DEMS measurement in which $Ni(OH)_2$ was used as the working electrode biased at a constant current density equivalent to 150 mA cm^{-2} of the cell.

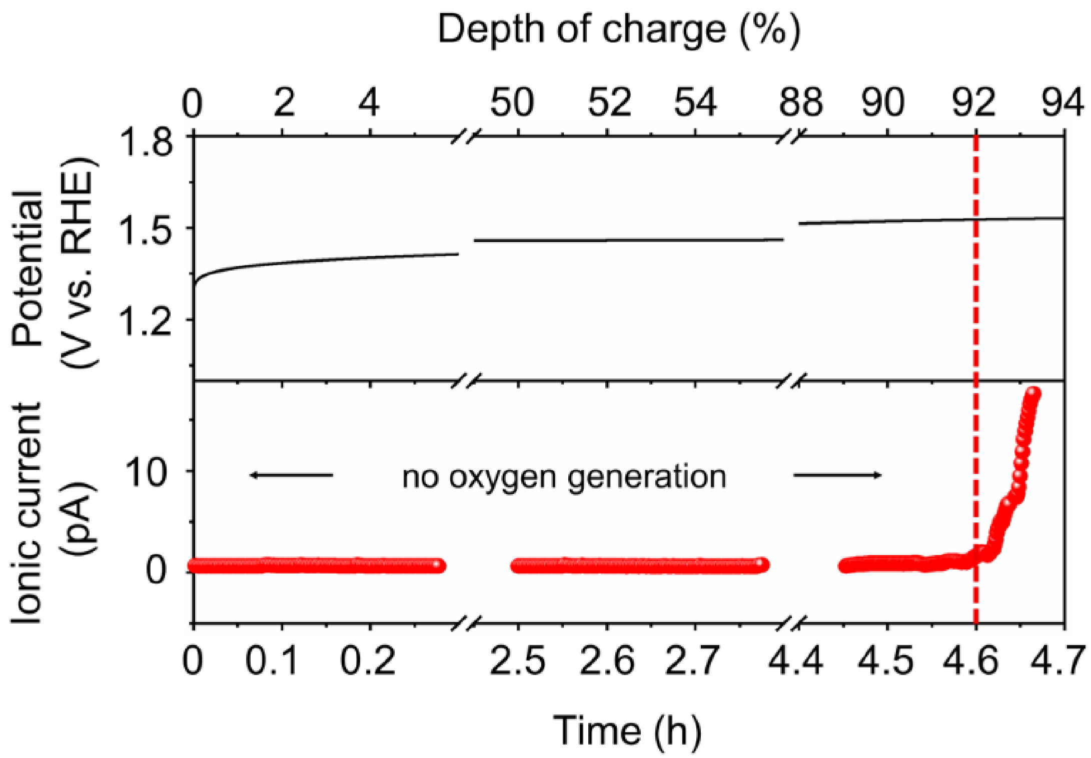

Supplementary Fig. 27. Monitoring O_2 production by DEMS. The ionic current response in the DEMS measurement in which $Ni(OH)_2$ was used as the working electrode biased at a constant current density equivalent to 40 mA cm^{-2} of the cell.

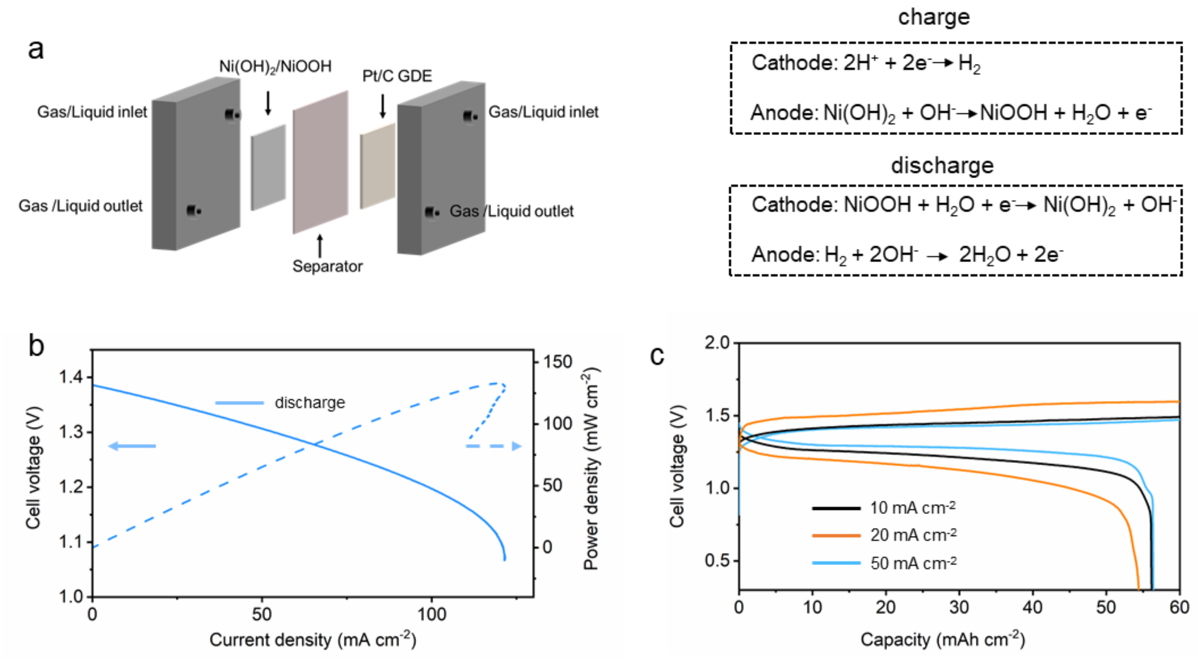

Supplementary Fig. 28. The performance of a home-built Ni-H_2 battery. (a) Schematic structure representation of Ni-H_2 battery; (b) I-V and I-P curves of the battery; (c) galvanostatic charge-discharge curves of the battery at different currents. The electrolyte was 1 M KOH.

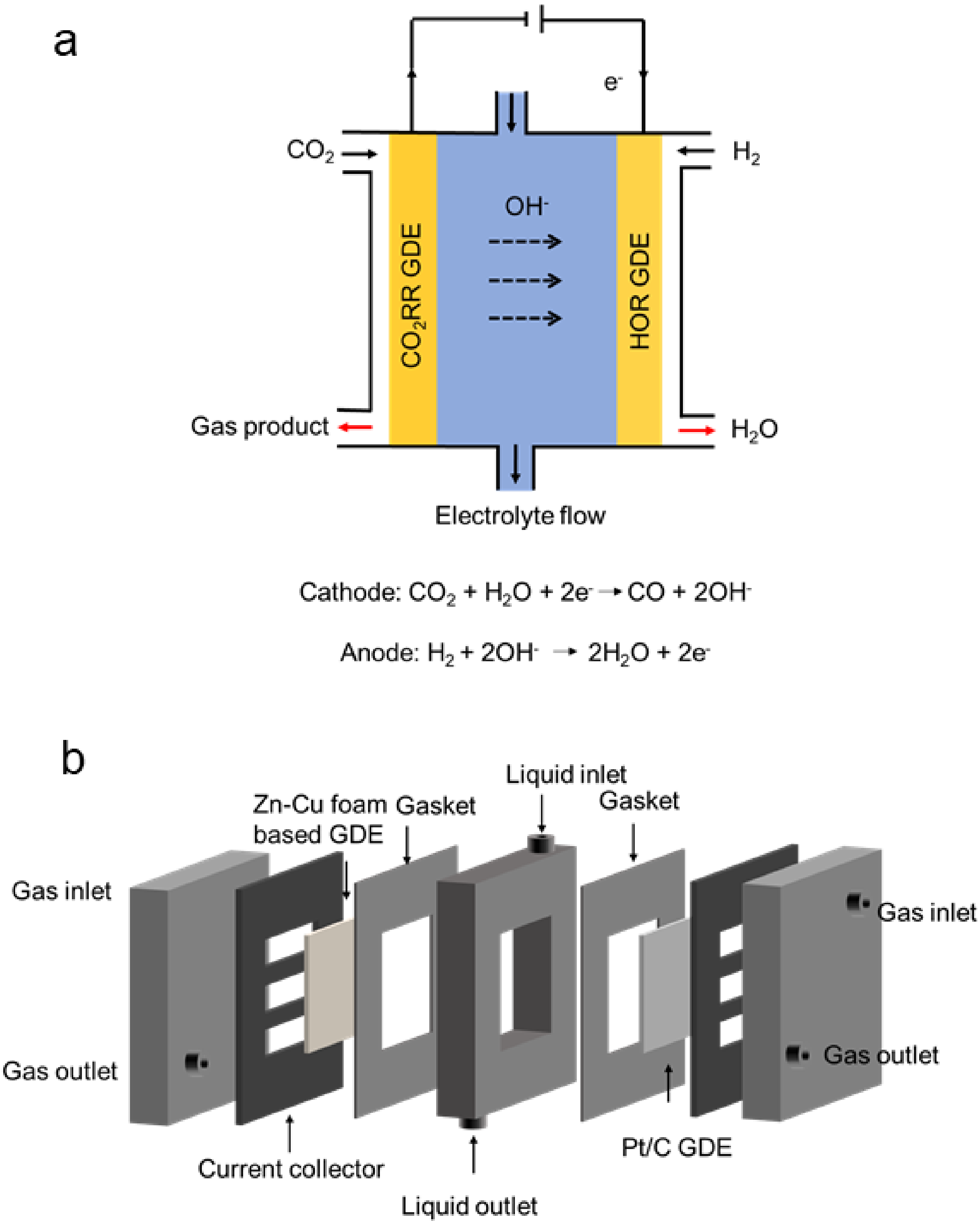

Supplementary Fig. 29. The cell structure for direct pairing of CO_2RR with HOR. (a) The working principle of CO_2RR coupled with the anodic HOR directly; (b) schematic representation of the cell configurations for CO_2RR with HOR occurring on the anode directly.

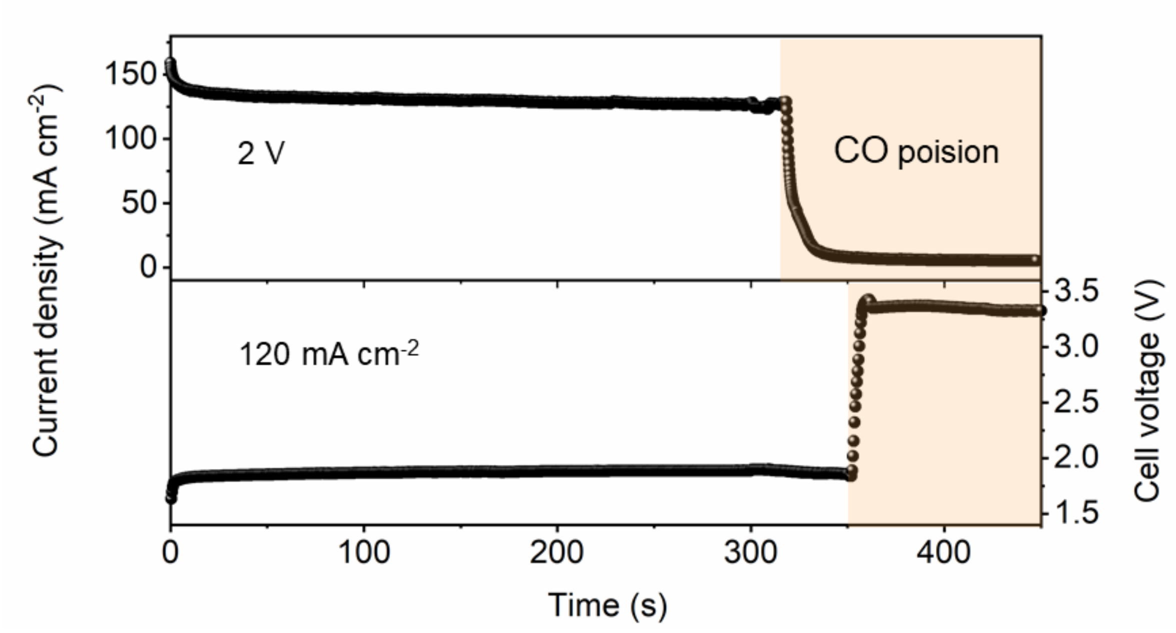

Supplementary Fig. 30. I-t and V-t curves of CO_2RR coupled with HOR occurring at the anode simultaneously in the cell.

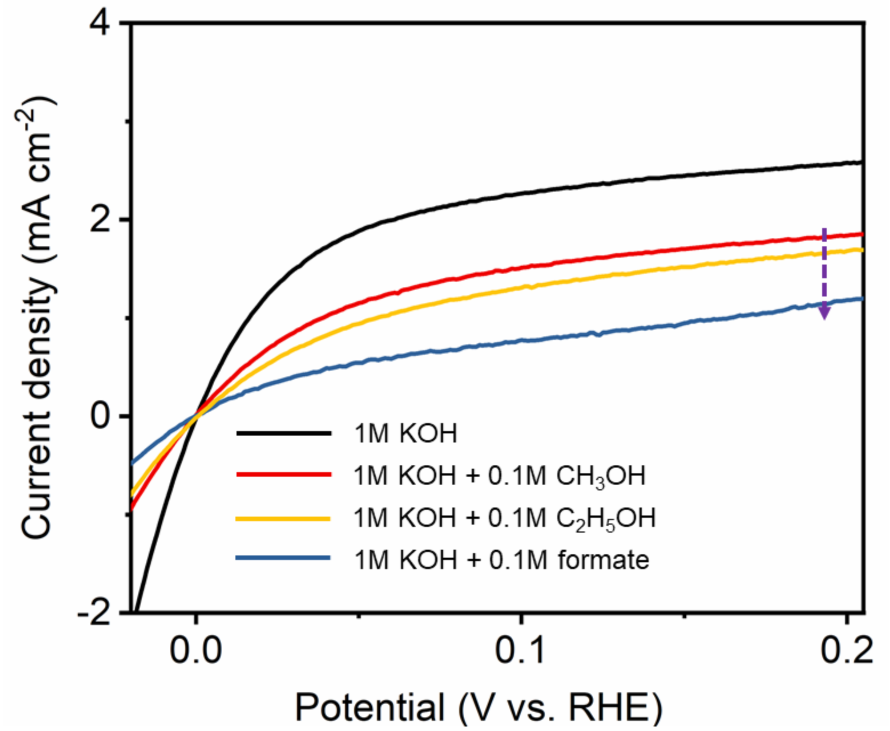

Supplementary Fig. 31. **The influence of typical CO_2RR product on the HOR activity of Pt/C electrode.** HOR polarization curves of Pt/C in 1 M KOH, 1 M KOH + 0.1 M CH_3OH, 1 M KOH + 0.1 M C_2H_5OH, and 1 M KOH + 0.1 M formate.

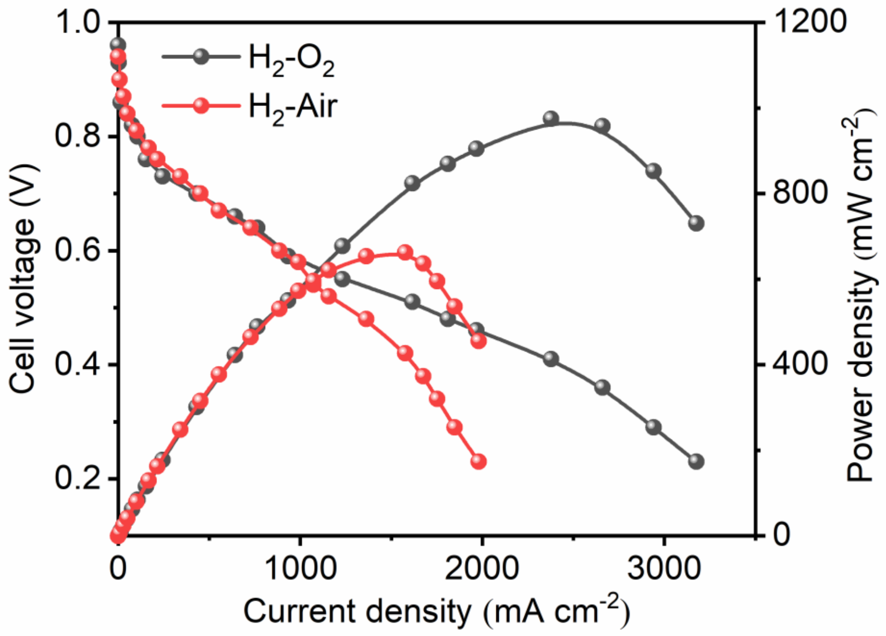

Supplementary Fig. 32. PEMFC performance. Polarization and power density plots of H_2-O_2 and H_2-air PEMFC.

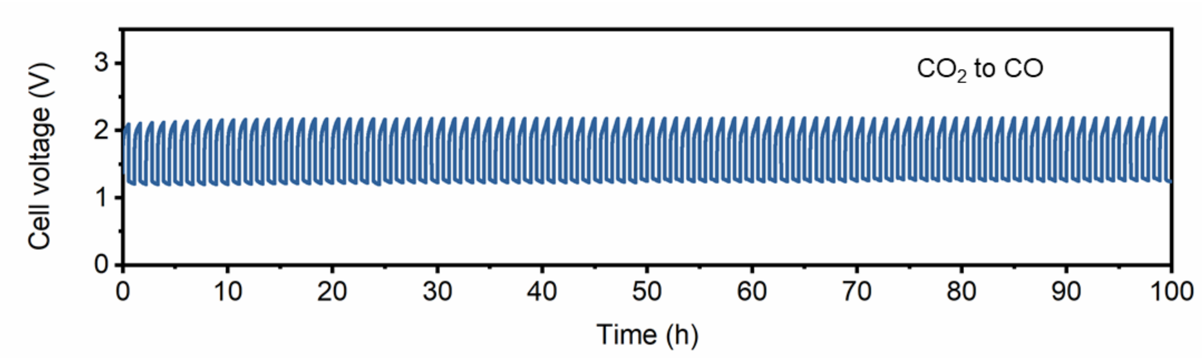

Supplementary Fig. 33. Longevity test of H_2-integarated CO_2RR combining periodical swap between Step 1 and Step 2 at 50 mA cm^{-2} for CO production.

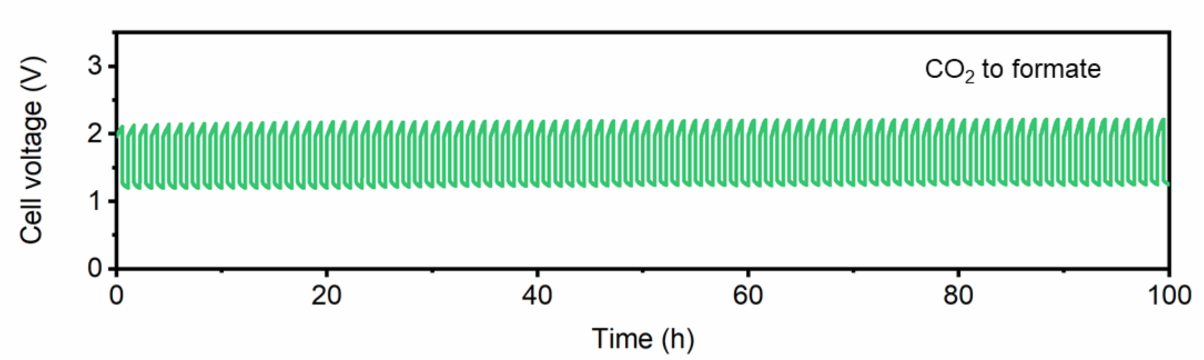

Supplementary Fig. 34. Longevity test of H_2-integarated CO_2RR combining periodical swap between Step 1 and Step 2 at 50 mA cm^{-2} for formate production.

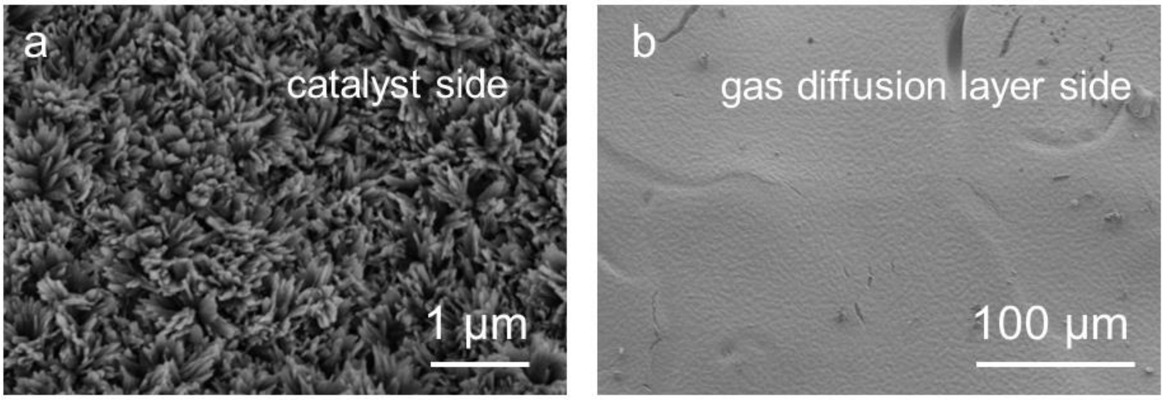

Supplementary Fig. 35. Characterizations of spent GDE. SEM images of Zn-Cu foam based GDE at the (a) catalysts side and (b) gas diffusion layer side after longevity test.

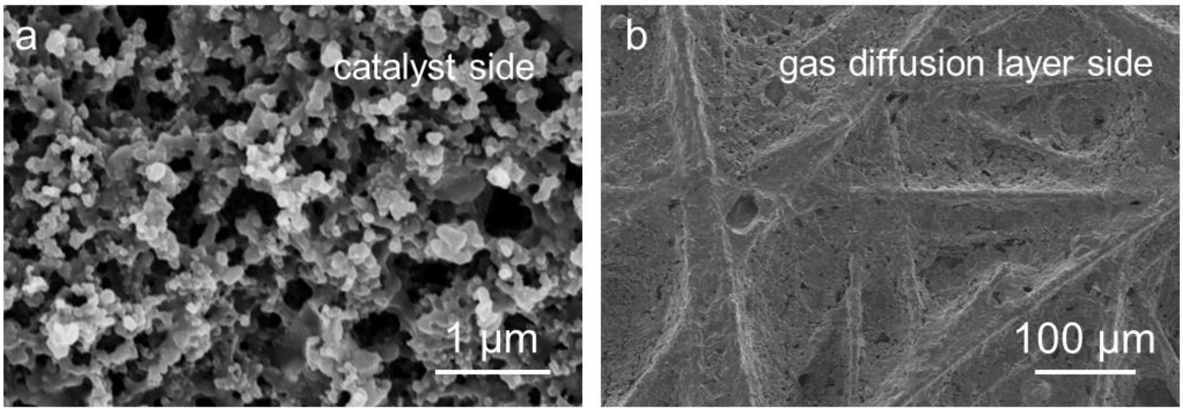

Supplementary Fig. 36. Characterizations of spent GDE. SEM images of Bi_2O_3-based GDE at the (a) catalysts side and (b) gas diffusion layer side after longevity test.

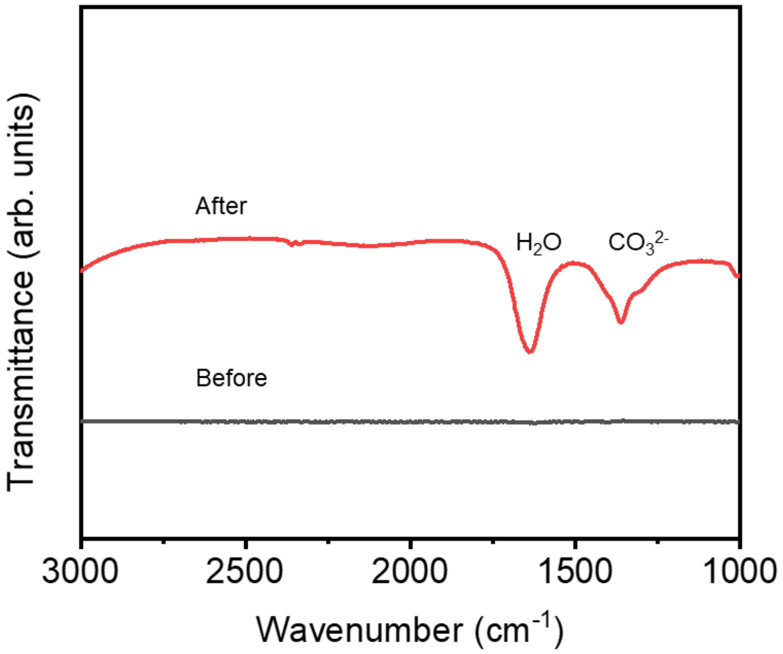

Supplementary Fig. 37. FTIR spectra of the Pt/C GDE before and after the operation in the H_2-integrated CO_2RR for the generation of CO.

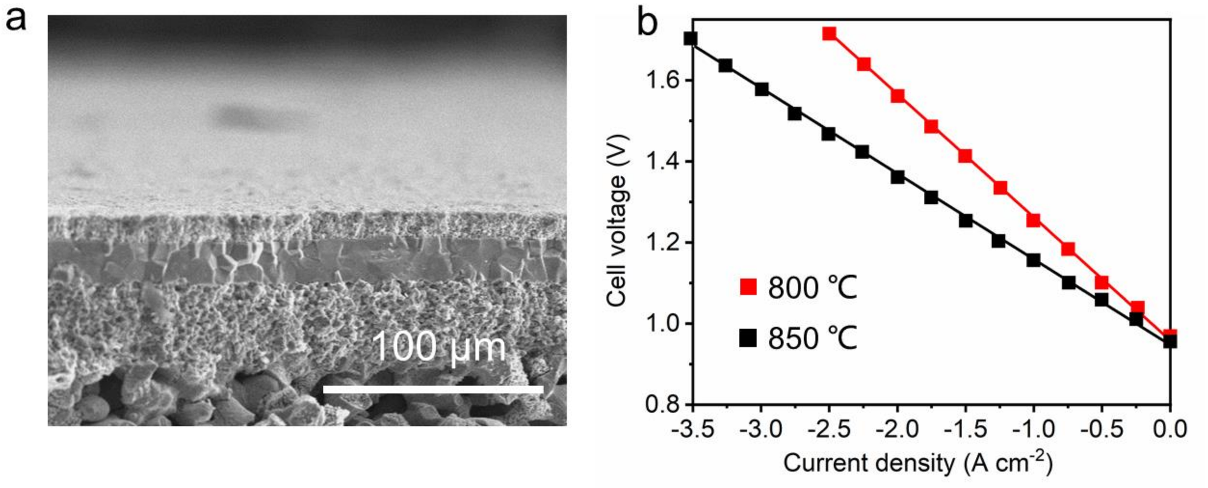

Supplementary Fig. 38. The characterizations and performance of SOEC. (a) cross-sectional SEM image of the electrode-supported SOEC; (b) polarization plots of water electrolysis at 800 °C and 850 °C, the inlet feedstock is 50 vol% H_2O balanced by H_2.

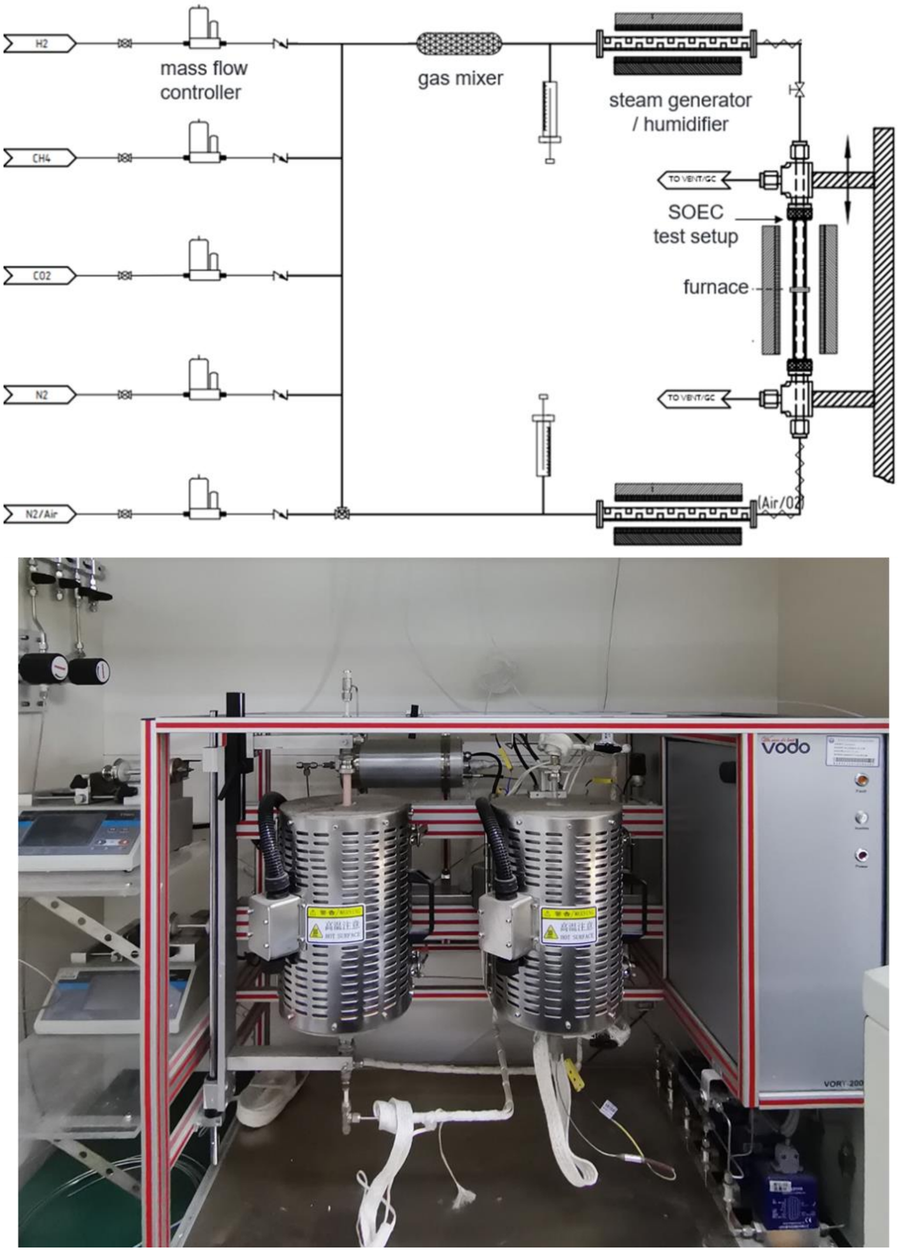

Supplementary Fig. 39. The design and photograph of a home-built solid oxide cells (SOC) test system for both SOEC and solid oxide fuel cells (SOFC) test.

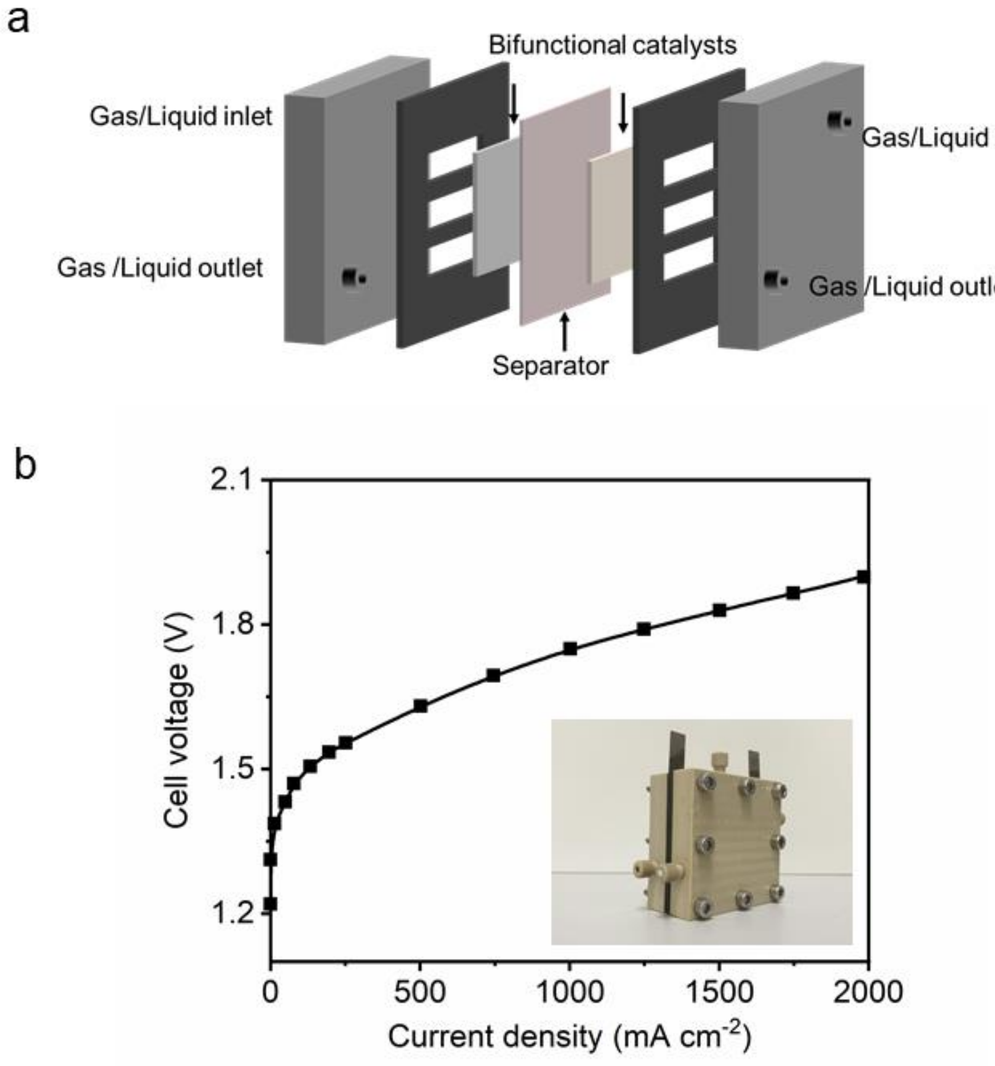

Supplementary Fig. 40. The cell structure and performance of alkaline water electrolyzer (AWE). (a) Schematic structure of a home-built AWE; (b) the polarization plot of water electrolysis in 6M KOH at 85 ℃ and ambient pressure.

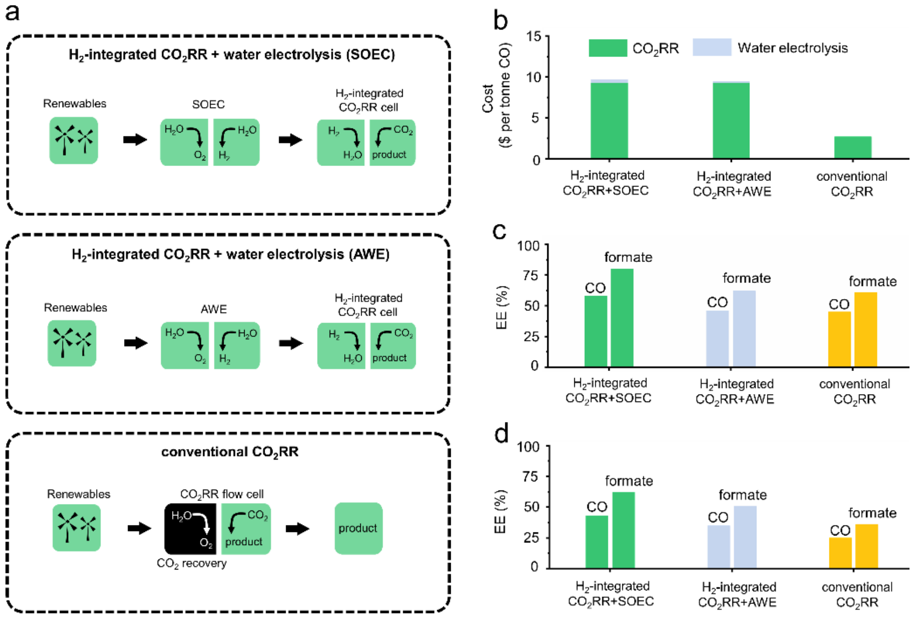

Supplementary Fig. 41. Techno-economic and energy efficiency analysis of H_2-integrated CO_2RR coupled with water electrolysis. (a) Schematic illustration of H_2-integrated CO_2RR coupled with water electrolysis and conventional CO_2RR; (b) preliminary techno-economic analysis based on CO production for H_2-integrated CO_2RR coupled with water electrolysis and conventional CO_2RR, only materials cost was considered; comparation of energy efficiency between H_2-integrated CO_2RR coupled with water electrolysis and conventional CO_2RR at 50 mA cm^{-2} in (c) alkaline and (d) neutral electrolyte.

3. Supplementary Notes

Supplementary Note 1: Calculation of voltage efficiency

The voltage efficiency of CO_2RR (Step 1 or conventional) was calculated as follows:

$$Voltage\ efficiency_1 = \frac{E_0}{E_c} \times 100\% \quad \text{Supplementary Equation (1)}$$

where E_c is the measured cell voltage, E_0 is the Nernst potential at standard conditions.

The voltage efficiency of "H_2-electricity" conversion (Step 2 or fuel cell) was calculated as follows:

$$Voltage\ efficiency_2 = \frac{E_c}{E_0} \times 100\% \quad \text{Supplementary Equation (2)}$$

For Step 2 in our cell, E_0 equals to 1.35 V. And for conventional H_2 fuel cell, E_0 equals to 1.23V.

Supplementary Note 2: Energy consumption analysis

We carried out the energy consumption analysis (GJ per tonne product) to evaluate the energy efficiency of H_2-integrated CO_2RR coupled with representative water electrolysis technologies (SOEC or AWE) in comparison with the conventional CO_2RR. The schematic comparison of three systems is shown in Supplementary Fig. 41. All values were obtained from the polarization curve of the corresponding electrochemical device. The total energy consumption for a specific process may comprise the following components:

(1) CO_2RR. The energy consumption of CO_2RR could be expressed as follows:

$$W[J] = E_c[V] \times \frac{Q\ [C]}{FE_{product}} \quad \text{Supplementary Equation (3)}$$

where E_c is the cell voltage at the selected current density; Q is the consumed electric charge when 1 tonne product was produced; $FE_{product}$ is the Faradaic efficiency of the desired product.

(2) SOEC. The total electric charge consumed in the production of H_2 equals to that consumed in the H_2-integrated CO_2RR cell. Thus, the energy consumption of SOEC at a given condition could be calculated via equation (3) where Q becomes the total charge.

(3) AWE. The method is identical with that of calculating SOEC energy consumption.

(4) Anodic CO_2 recovery. At pH=7, the energy consumption of anodic CO_2 recovery must be considered. The CO_2 crossover rate at the anode is proportional to the current. We assumed that 0.5 moles of CO_2 is lost per mole of electron transferred based on the literatures[19-20, 31]. Thus, the amount of evolved CO_2 at the anode could be determined using the following

equation:

$$Theoritical\ CO_2\ crossover\ [\text{mol}] = 0.5 \times \frac{Q[C]}{F[\frac{C}{mol}] \times FE_{product}} \quad \text{Supplementary Equation (4)}$$

we assume the CO_2 capture cost, via amine scrubbing, is 4.0 GJ per tonne CO_2[32-34]. CO_2 loss at the cathode and in the electrolyte was not considered.

The energy efficiency (EE) of each process could be expressed as follows:

$$EE = \frac{n_j \times LHV_j}{W_{consumption}} \times 100\% \quad \text{Supplementary Equation (5)}$$

where $W_{consumption}$ is the total energy consumption, including that of water electrolysis when applicable, for each route; LHV_j is the lower heating value of product (CO: 282.98 kJ mol^{-1}; HCOOH: 209.82 kJ mol^{-1}); and n_j is the molar amount of product.

Supplementary Note 3: Techno-economic analysis

The techno-economic analysis of three processes was also evaluated based on an established model in the literature assuming a pilot plant producing 100 tonne of CO per day[35]. To simplify our calculation model, we only consider the material cost as the operational cost of the new process is challenging to quantify accurately. The materials cost was calculated via the following equation:

$$C_{materials}[\frac{\$}{\text{kW}}] = \frac{\sum_n p_n A_n[\$]}{P[\text{kW}]} \quad \text{Supplementary Equation (6)}$$

where p is the operation power of the cell, p_n is unit price of the cell component n, and A_n is the area of each cell component. Details are shown below.

H_2-integrated CO_2RR + SOEC. The total cost for H_2-integrated CO_2RR + SOEC is ~\$9.7 per tonne CO which comprises of:

(1) Step 1 of H_2-integrated CO_2RR. The cell components contain Zn-Cu foam GDE and $Ni(OH)_2$/NiOOH electrode (the unit prices were shown in Supplementary Table 20). We selected the power at 50 mA cm^{-2} (2.15 V, 0.11 W cm^{-2}) as a reference. Thus, the calculated materials cost of Step 1 was ~\$64 kW^{-1}. The total equivalent current needed to produce 100 tonne CO per day can be obtained via Equation (7) assuming 100% CO FE.

$$Total\ current\ needed\ [\text{A}] = \frac{mole\ number\ of\ CO\ production\ [\frac{\text{mol}}{\text{day}}] \times electrons\ transfer \times F[\frac{\text{C}}{\text{mol}}]}{24 \times 3600\ [\frac{\text{s}}{\text{day}}]}$$

Supplementary Equation (7)

The total equivalent current needed was thus 7976604 A. The required power was 17149.7 kW using the following equation:

$$Power\ consumed\ [\mathrm{kW}] = Total\ current\ needed\ [\mathrm{A}] \times cell\ voltage\ [\mathrm{V}]$$

Supplementary Equation (8)

Then, the total materials cost of Step 1 was $1097580 using the following equation:

$$Total\ materials\ cost\ [\$] = Power\ Consumed\ [\mathrm{kW}]\ \times\ Electrolyzer\ Cost\ [\$\ \mathrm{kW}^{-1}]\ (9)$$

This cost refers to the overall one-time investment cost of materials, which needs to be translated into a cost per tonne of CO. Turning a long-term investment into a daily cost is based on a capital recovery factor (CRF) defined as follows:

$$CRF = \frac{i(1+i)^{lifetime}}{(1+i)^{lifetime}-1}$$

Supplementary Equation (10)

where i is a discount rate assumed to be 7% [35]; the lifetime of 5 years is used for all materials and all conditions. Thus, CRF was calculated to be 0.24389.

The final materials cost can be expressed as:

$$materials\ cost\ \left[\frac{\$}{\mathrm{tonne\ CO}}\right] = \frac{CRF \times total\ materials\ cost\ [\$]}{capacity\ factor\ \left[\frac{\mathrm{days}}{\mathrm{year}}\right] \times production\ \left[\frac{\mathrm{tonne\ CO}}{\mathrm{day}}\right]}$$

Supplementary Equation (11)

Assuming a plant capacity factor of 0.9, we found the material cost was $8.1 per tonne CO in the Step 1 of H_2-integrated CO_2RR.

(2) Step 2 of H_2-integrated CO_2RR. We only considered Pt GDE and separator, as other components were considered in Step 1. The selected the maximum power density of 0.22 W cm^{-2}. Thus, the calculated materials price for Step 2 was ~$15.4 kW^{-1}. Because the consumed charge in Step 1 and Step 2 is identical, the total equivalent current needed here was the same as that in Step 1. Using equations (8)-(11), the materials cost was ~$1.2 per tonne CO.

(3) SOEC. The cell contains cathode (NiO and YSZ), YSZ electrolyte and anode ($La_{0.8}Sr_{0.2}MnO_{3-x}$). All unit prices were shown in Supplementary Table 20. The power density was selected at 2 A cm^{-2} as a reference (1.34 V, 2.68 W cm^{-2}, see the polarization plot at 850 °C in Supplementary Fig. 38). Thus, the calculated cost was ~$5.1 kW^{-1}, or $0.4 per tonne CO.

H_2-integrated CO_2RR + AWE. The total cost for H_2-integrated CO_2RR + AWE is ~$9.5 per tonne CO which comprises of

(1) H_2-integrated CO_2RR cell. See calculation details above.

(2) AWE. The AWE cell contains two Co-Ni phosphide/spinel oxide hybrid bifunctional electrodes, and a piece of separator (the unit prices were shown in Supplementary Table 20). We selected the typical working condition of 500 mA cm^{-2} as a reference (1.63V, 0.3 W cm^{-2}, see the polarization plot in Supplementary Fig. 40). Thus, the calculated materials cost was ~\$1.9 kW^{-1}, or \$0.2 per tonne CO.

Conventional CO_2RR. The cell components contain Zn-Cu foam GDE and Co_3O_4-Ni electrode (the unit prices were shown in Supplementary Table 20). The selected power was also at 50 mA cm^{-2} (2.32V, 0.12 W cm^{-2}). Thus, the calculated cost was ~\$19 kW^{-1}, or \$2.7 per tonne CO.

4. Supplementary References

1. Ren, S. et al. Molecular electrocatalysts can mediate fast, selective CO_2 reduction in a flow cell. *Science* **365**, 367-369 (2019).
2. Reyes, A. et al. Managing hydration at the cathode enables efficient CO_2 electrolysis at commercially relevant current densities. *ACS Energy Lett.* **5**, 1612-1618 (2020).
3. Zheng, T. et al. Large-Scale and Highly Selective CO_2 Electrocatalytic Reduction on Nickel Single-Atom Catalyst. *Joule*, 3 (2019).
4. Kim, D. et al. Electrocatalytic reduction of low concentrations of CO_2 Gas in a membrane electrode assembly electrolyzer. *ACS Energy Lett.* **6**, 3488-3495 (2021).
5. Lim, C. et al. Enhancement of catalytic activity and selectivity for the gaseous electroreduction of CO_2 to CO: guidelines for the selection of carbon supports. *Adv. Sustain. Syst.* **5**, 2100216 (2021).
6. Verma, S., Lu, S.&Kenis, P. J. A. Co-electrolysis of CO_2 and glycerol as a pathway to carbon chemicals with improved technoeconomics due to low electricity consumption. *Nat. Energy* **4**, 466-474 (2019).
7. Wei, X., Li, Y., Chen, L.&Shi, J. Formic acid electro-synthesis by concurrent cathodic CO_2 reduction and anodic CH_3OH oxidation. *Angew. Chem. Int. Ed.* **60**, 3148-3155 (2021).
8. Wang, G. et al. Cost-effective and durable electrocatalysts for co-electrolysis of CO_2 conversion and glycerol upgrading. *Nano Energy* **92**, 106751 (2022).
9. Medvedeva, X. V. et al. Sustainable at both ends: electrochemical CO_2 utilization paired with electrochemical treatment of nitrogenous waste. *Green. Chem* **14**, 4456-4462 (2020).
10. Pei, Y. et al. Glycerol oxidation-assisted electrochemical CO_2 reduction for the dual production of formate. *J. Mater. Chem. A* **10**, 1309-1319 (2022).
11. Guo, J.-H.&Sun, W.-Y. Integrating nickel-nitrogen doped carbon catalyzed CO_2 electroreduction with chlor-alkali process for CO, Cl_2 and $KHCO_3$ production with enhanced techno-economics. *Appl. Catal. B: Environ.* **275**, 119154 (2020).
12. Bi, J. et al. Simultaneous CO_2 reduction and 5-hydroxymethylfurfural oxidation to value-added products by electrocatalysis. *ACS Sustain. Chem. Eng.* **10**, 8043-8050 (2022).
13. Ge, R. et al. Intensified coupled electrolysis of CO_2 and brine over electrocatalysts with

ordered mesoporous transport channels. *Chem. Eng. J.* **438**, 135500 (2022).
14. Xiao, C. et al. Low full-cell voltage driven high-current-density selective paired formate electrosynthesis. *J. Mater. Chem. A* **10**, 1329-1335 (2022).
15. Pei, Y., Wang, C., Zhong, H.&Jin, F. Concurrent electrolysis under pressured CO_2 for simultaneous CO_2 reduction and hazardous SO_2 removal. *ACS Sustain. Chem. Eng.* **10**, 12670-12678 (2022).
16. Liang, Y. et al. Unveiling in situ evolved In/In(2)O(3-)(x) heterostructure as the active phase of In(2)O(3) toward efficient electroreduction of CO(2) to formate. *Sci. Bull.* **65**, 1547-1554 (2020).
17. Ma, W. et al. Achieving simultaneous CO_2 and H_2S conversion via a coupled solar-driven electrochemical approach on non-precious-metal catalysts. *Angew. Chem. Int. Ed. Engl.* **57**, 3473-3477 (2018).
18. Hu, Y. et al. Coplanar Pt/C nanomeshes with ultrastable oxygen reduction performance in fuel cells. *Angew. Chem. Int. Ed.* **60**, 6533–6538 (2021).
19. Liu, X. et al. Inducing covalent atomic interaction in intermetallic Pt alloy nanocatalysts for high-performance fuel cells. *Angew. Chem. Int. Ed. Engl.*, e202302134 (2023).
20. Wan, X. et al. Fe–N–C electrocatalyst with dense active sites and efficient mass transport for high-performance proton exchange membrane fuel cells. *Nat. Catal.* **2**, 259-268 (2019).
21. Zhang, G. et al. Non-PGM electrocatalysts for PEM fuel cells: effect of fluorination on the activity and the stability of a highly active NC_Ar+NH3 catalyst. *Energy Environ. Sci.* **12**, 3015-3037 (2019).
22. Xia, D. et al. Ultrastable Fe-N-C fuel cell electrocatalysts by eliminating non-coordinating nitrogen and regulating coordination structures at high temperatures. *Adv. Mater.* **35**, e2204474 (2023).
23. Muller-Hulstede, J. et al. Towards the reduction of Pt loading in high temperature proton exchange membrane fuel cells - effect of Fe-N-C in Pt-alloy cathodes. *ChemSusChem* **16**, e202202046 (2023).
24. Adjemian, K. T. et al. Function and characterization of metal oxide-nafion composite membranes for elevated-temperature H_2/O_2 PEM fuel vells. *Chem. Mater.* **18**, 2238-2248 (2006).
25. Deng, Y. et al. Hierarchically open-porous carbon networks enriched with exclusive Fe–Nx active sites as efficient oxygen reduction catalysts towards acidic H_2–O_2 PEM fuel cell and alkaline Zn–air battery. *Chem. Eng. J.* **390**, 124479 (2020).
26. Tang, H. et al. Properties and stability of quaternary ammonium-biphosphate ion-pair poly(sulfone)s high temperature proton exchange membranes for H_2/O_2 fuel cells. *J. Power Sources* **475**, 228521 (2020).
27. Chen, W. et al. Nickel-hydrogen batteries for large-scale energy storage. *Proc. Natl. Acad. Sci. U.S.A.* **115**, 11694-11699 (2018).
28. Singh, N.&McFarland, E. W. Levelized cost of energy and sensitivity analysis for the hydrogen–bromine flow battery. *J. Power Sources* **288**, 187-198 (2015).
29. Li, Z.&Lu, Y.-C. Polysulfide-based redox flow batteries with long life and low levelized cost enabled by charge-reinforced ion-selective membranes. *Nat. Energy* **6**, 517-528 (2021).
30. Pribyl-Kranewitter, B. et al. Influence of low-temperature electrolyser design on economic

and environmental potential of CO and HCOOH production: A techno-economic assessment. *Renew. Sust. Energ. Rev.* **154**, 111807 (2022).
31. Ma, M. et al. Insights into the carbon balance for CO_2 electroreduction on Cu using gas diffusion electrode reactor designs. *Energy Environ. Sci.* **13**, 977-985 (2020).
32. Xie, K. et al. Eliminating the need for anodic gas separation in CO_2 electroreduction systems via liquid-to-liquid anodic upgrading. *Nat. Commun.* **13**, 3070 (2022).
33. Alerte, T. et al. Downstream of the CO_2 electrolyzer: assessing the energy intensity of product separation. *ACS Energ. Lett.* **6**, 4405-4412 (2021).
34. Boot-Handford, M. E. et al. Carbon capture and storage update. *Energy Environ. Sci.* **7**, 130-189 (2014).
35. Sisler, J. et al. Ethylene electrosynthesis: a comparative techno-economic analysis of alkaline vs membrane electrode assembly vs CO_2–CO–C_2H_4 tandems. *ACS Energ. Lett.* **6**, 997-1002 (2021).
